# Stepwise establishment of functional microbial groups in the infant gut between 6 months and 2 years: A prospective cohort study

**DOI:** 10.3389/fnut.2022.948131

**Published:** 2022-07-28

**Authors:** Van T. Pham, Anna Greppi, Christophe Chassard, Christian Braegger, Christophe Lacroix

**Affiliations:** ^1^Laboratory of Food Biotechnology, Institute of Food, Nutrition and Health, ETH Zurich, Zurich, Switzerland; ^2^Division of Gastroenterology and Nutrition, University Children's Hospital Zurich, Zurich, Switzerland

**Keywords:** infant gut microbiota, lactate utilization, colonization, functional community, butyrate-producing bacteria

## Abstract

The early intestinal colonization of functional microbial groups plays an essential role in infant gut health, with most studies targeting the initial colonization period from birth to 6 months of age. In a previous report, we demonstrated the metabolic cross-feeding of lactate and identified keystone species specified for lactate utilization in fecal samples of 40 healthy infants. We present here the extension of our longitudinal study for the period from 6 months to 2 years, with a focus on the colonization of functional groups involved in lactate metabolism and butyrate production. We captured the dynamic changes of the gut microbiota and reported a switch in the predominant lactate-producing and lactate-utilizing bacteria, from *Veillonella* producing propionate in the first year to *Anaerobutyrycum hallii* producing butyrate in the second year of life. The significant increase in butyrate producers and fecal butyrate concentration was also pinpointed to the weaning period between 6 and 10 months. Correlation analyses further suggested, for the first time, the metabolic cross-feeding of hydrogen in infants. In conclusion, our longitudinal study of 40 Swiss infants provides important insights into the colonization of functional groups involved in lactate metabolism and butyrate production in the first 2 years of life.

## Introduction

The greatest influence on the development and establishment of the gut microbiota occurs at birth when the infant is exposed to vaginal, fecal, and skin microbiota from the mother and to microbes from the environment (1–5). A growing body of evidence demonstrated that the establishment of intestinal microbiota in infancy significantly influences health later in life. Disturbed colonization may adversely affect the gut development of host defense and predispose to inflammation, leading to increased susceptibility to diseases, such as allergy, autoimmune disease, overweight, atopic sensibilization, etc., later in life (6).

The most effective way to explore the gut colonization process in infants is through longitudinal studies, with a focus on specific time windows such as from birth to 6 months and from 6 months to 2 years of age. In such a cohort study covering the first months of life, we demonstrated the early predominance of strict anaerobes which outnumbered facultative anaerobes within the first week of life (7). The switch from infant to adult-associated profile is then observed between 1 and 2 years old (8), indicating that critical changes in the gut microbiome occur in this time window. Among others, such microbiome shifts are consistently associated with life events such as the cessation of breastfeeding and the introduction of solid food, and the use of antibiotics (9–13).

In the infant's gut, several microbial functional groups work in harmony in a so-called trophic chain to metabolize different sources of carbohydrates reaching the colon and form end metabolites, mainly acetate and propionate (14–18). Most primary colonizers in the infant's gut are lactate-producing bacteria (LPB), including bifidobacteria, lactobacilli, and *Bacteroides* (4, 7, 17, 19). Lactate is produced in large amounts in the infant's gut and must be re-used by lactate-utilizing bacteria (LUB) to avoid detrimental consequences of lactate accumulation, such as metabolic acidosis (20, 21). We investigated the colonization of LPB and LUB and emphasized the importance of cross-feeding of lactate while identifying keystone LUB species in the first 6 months of life (15, 17). The metabolism of lactate might play a key role in the etiology of gastrointestinal symptoms, such as infant colic, a functional gastrointestinal disorder that affects up to 20% of infants. Indeed, we detected specific LUB signatures between colicky and non-colicky infants and for crying and non-crying infants, suggesting that a decrease in H_2_ utilization by LUB sulfate-reducing bacteria (SRB) and an increase in H_2_ production by LUB non-SRB could lead to acute H_2_ accumulation associated with crying and infantile colic (22, 23). However, the development of those functional groups from 6 to 24 months of life when the infant gut microbiota undergoes a major transition toward an adult-associated profile remained largely unexplored. Moreover, butyrate has been identified as a major energy source for colonocytes and exerts anti-inflammatory and anticancer properties (24–27). However, little is known about the prevalence and establishment of butyrate-producing bacteria (BPB) in infants from 6 to 24 months.

Therefore, the aim of the present study was to investigate the colonization patterns of gut microbiota of 40 infants from 6 months to 2 years of life, focusing on functional microbial groups involved in lactate metabolism and butyrate production. Our longitudinal study investigated the prevalence, levels, and relative abundances of functional bacterial groups using a combination of cultural and molecular methods. Metabolic activity of the gut microbiota was assessed indirectly in feces by high-performance liquid chromatography (HPLC). Our results shed light on the lactate and H_2_ cross-feeding by analyzing correlations between LPB, LUB, BPB, and short-chain fatty acids (SCFA). Furthermore, with our data, we explored further the impact of mode of delivery and infant colic on the infant gut microbiome of infants from 6 months to 2 years.

## Materials and methods

This study is an extension of a previous report of the same infant cohort covering the first 6 months of life (15).

### Study population

Fifty-six mothers-to-be were contacted for participation at the University Children's Hospital (Zurich, Switzerland) as described previously (15). Thirteen women did not consent to participate in the study or were excluded due to gastrointestinal or immunological preexisting conditions. Forty-three healthy term infants born without congenital diseases were enrolled in this study. Three infants dropped out during the follow-up period and were not included in the final analysis. No infant was excluded based on the exclusion criteria, i.e., variables known to affect the balance of the gut microbiota, including pre-term birth, gastrointestinal, and immunological disorders during the neonatal period. Informed written consent was obtained from all participants. The study was approved by the Ethics Committee of ETH Zurich (Project EK 2012-N-36; date of approval 28.09.2012). Questionnaires were collected from all participating infants. General characteristics, antibiotic usage, and feeding practice of this cohort are described in Supplementary Table 1.

### Fecal sample collection

**Figure 1:**
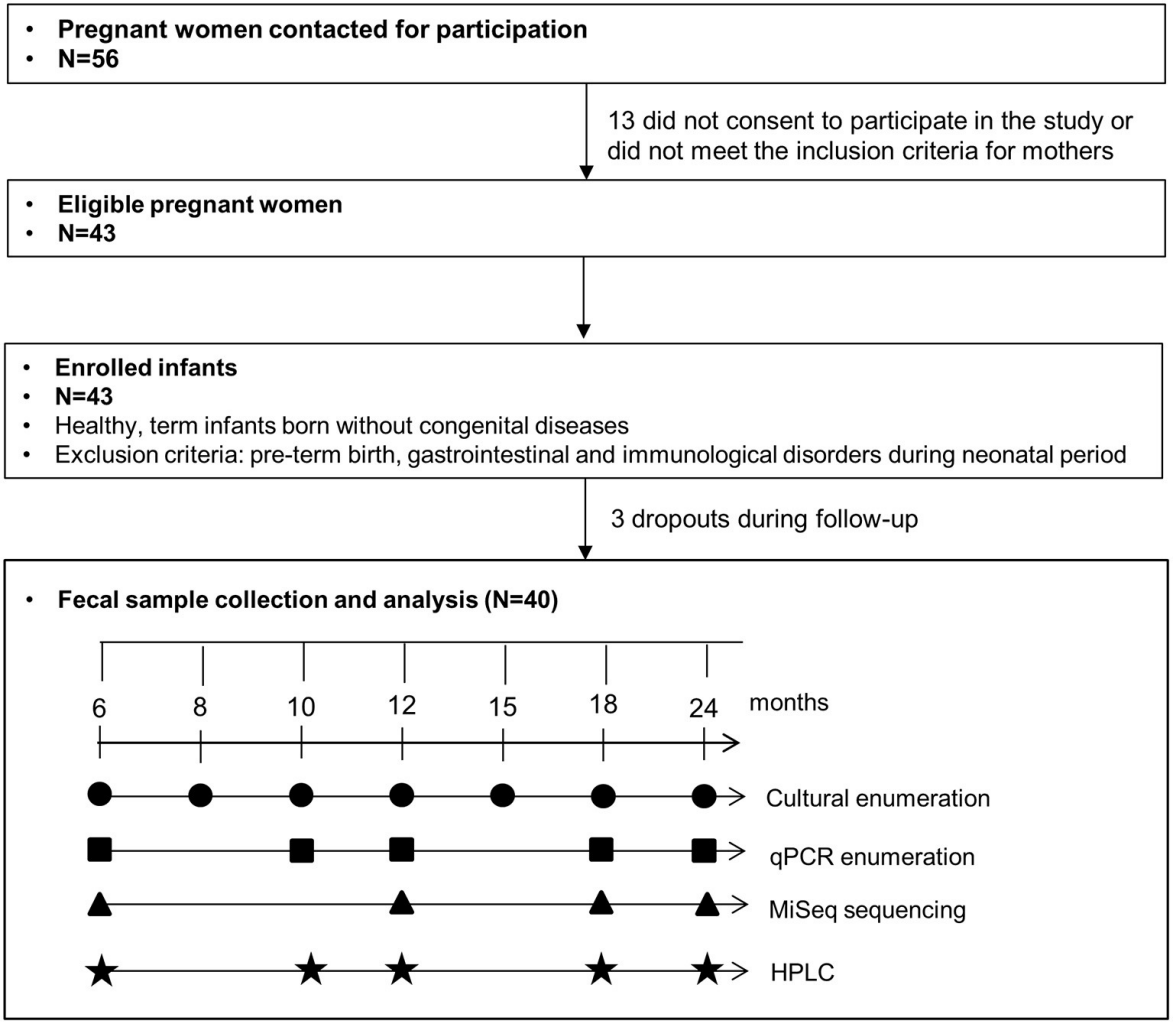
Sampling and analysis scheme of this study.

Infant fecal samples were collected at seven sampling points from 6 to 24 months with the analysis scheme presented in Figure 1. Fresh infant feces were collected from diapers, transferred into a fecal collection container, and transported under anaerobiosis at 4°C until processing of samples within 8 h at the laboratory. Immediately upon receipt, aliquots were prepared for culture-based enumeration in an anaerobic chamber. Aliquots were stored at −80°C before DNA extraction for qPCR and sequencing.

### DNA extraction and quantification

For Illumina MiSeq sequencing analysis, total genomic DNA was extracted from 200 mg of fresh infant feces using the QIAamp DNA Stool Mini Kit (Qiagen, Hilden, Germany) according to the manufacturer's instructions. Samples were stored for 2 weeks at 4°C prior to sequencing analysis. For quantitative PCR, total genomic DNA was extracted from 200 mg of fresh infant feces using the Fast DNA SPIN kit for soil (MP Biomedicals, Illkirch, France) according to the manufacturer's instructions. DNA concentration and quality were accessed by absorbance measurements at 260 nm on a NanoDrop^®^ ND-1000 Spectrophotometer (Witec AG, Littau, Switzerland), and samples were stored at −20°C before qPCR analysis.

### Amplicon sequencing

The microbiota community was analyzed in all fecal samples at 6, 12, 18, and 24 months. Illumina MiSeq sequencing analysis targeting V3-V4 hypervariable 16S rRNA subunit was carried out at Genotoul (Toulouse, France) using specific forward F340 (5′-CCTACGGRAGGCAGCAG-3′) and reverse primer R805 (5′- GGACTACHVGGGTWTCTAAT-3′). Thermocycling was performed with an initial step at 94°C for 60 s, followed by 30 cycles of denaturation at 94°C for 60 s, annealing at 65°C for 60 s, and elongation at 72°C for 60 s, with a final elongation of 10 min at 72°C. The raw dataset containing pair-ended reads with corresponding quality scores was merged and trimmed using settings as previously mentioned (28). Quantitative Insight Into Microbial Ecology (QIIME) open-source software package (1.8.0) was used for subsequent analysis steps. Purging the dataset from chimeric reads and constructing *de novo* Operational Taxonomic Units (OTU) was conducted using the UPARSE pipeline. The HIT 16S rRNA gene collection was used as a reference database.

### Enumeration of functional microbes by culture

All liquid media were prepared, dispensed, and inoculated using strict anaerobic techniques with 100% O_2_-free CO_2_ as outlined by Hungate (29). The culturable lactate-utilizing bacteria (LUB) community consisted of the lactate-utilizing sulfate-reducing bacteria (Cul LUB SRB) and the lactate-utilizing non-sulfate-reducing bacteria (Cul LUB non-SRB). The Cul LUB SRB and Cul LUB non-SRB community were enumerated at all timepoints as described previously (15), using the most probable number estimation in Postgate E and M2GSC medium, respectively.

### Quantitative PCR analysis

qPCR analysis was performed using an ABI PRISM 7500-PCR sequence detection system (Applied Biosystems, Zug, Switzerland) with 2 x KapaSybr Fast qPCR Mastermix (Biolabo Scientific Instruments, Châtel-St-Denis, Switzerland) and 1 μl template genomic DNA in a total volume of 25 μl. Amplification conditions were described previously (15). Specific primers targeting bacterial groups or species prevalent in the gut microbiota were used at a concentration of 0.2 μM and are presented in Supplementary Table 2. A dilution series of the standard was included in each run. Positive qPCR samples were defined as samples with the number of gene copies/μl higher than the number of gene copies/μl of the standard at the highest dilution that could generate good amplification and melting curve.

### Sequencing data analysis

Unweighted, generalized, and weighted UniFrac distance metrics were calculated from subsampled OTU tables (10,000 reads/sample) and visualized with PCA plots. Significant differences were tested using Permutational Multivariate Analysis of Variance using Distance Matrices, “adonis” function from R package “vegan.” Alpha diversity measures expressed with an observed species (sequence similarity 97% OTUs) value were computed for rarefied OTU tables (10,000 reads/sample) using the alpha rarefaction workflow. Differences in alpha diversity were determined using a *t*-test-based approach employing the non-parametric (Monte Carlo) method (999 permutations) implemented in the compare alpha diversity workflow. ANCOM (Analysis of Composition of Microbes) analysis was performed to detect significantly different taxa over time (30). Only taxa more abundant than 1% in the samples were considered for ANCOM analysis (*P* < 0.05).

### Fecal sample metabolic analysis

Lactose, glucose, lactate, SCFA (acetate, propionate, and butyrate), and branched-chain fatty acids (BCFA) (isobutyrate and isovalerate) were measured in fecal samples of 40 infants at 6, 10, 12, 18, and 24 months by HPLC as described previously (15). Fecal samples were mixed with 1 ml 0.15 mM H_2_SO_4_, homogenized, and centrifuged at 4°C at 9,000 × *g* for 20 min. Clear supernatants were passed through a 0.45 μl filter (Infochroma AG, Zug, Switzerland) before injection. HPLC analysis (Thermo Fisher Scientific Inc. Accela, Wohlen, Switzerland) was performed using a SecurityGuard Cartridges Carbo-H (4 ×3.0 mm) (Phenomenex, Torrance, CA, USA) connected to a Rezex ROA-Organic Acid H+ (300 × 7.8 mm) column (Phenomenex, Torrance, CA, USA) at a flow rate of 0.4 mL min^−1^ at 40°C and 10 mM H_2_SO_4_ as eluent solution. Data were expressed as μmol g^−1^ feces.

### Statistical analysis

For cultural, qPCR, and SCFA data, statistical analysis was done using IBM SPSS Statistics 20.0 (IBM SPSS Inc, Chicago, IL, USA). The normality of each dataset was assessed using Shapiro-Wilk tests. Cultural and qPCR data above the detection limit were log_10_ transformed. For qPCR data used for correlation and comparison analysis, default values of half the detection limit (Supplementary Table 2) were assigned for values below the threshold of the qPCR (see above). The tests revealed non-normal distribution, cultural, qPCR, and SCFA data were expressed as a median. The differences in median composition and metabolic changes across time points were assessed using non-parametric multiple comparisons with the Kruskal Wallis test. Changes in microbial levels enumerated by cultural and qPCR, and metabolite concentration over time were assessed using linear regression. Spearman correlation R and corresponding *q* values between metabolite concentrations and bacterial levels were calculated. *P* < 0.05 were considered significant unless otherwise stated.

## Results

### Microbiota composition profile of infants' fecal samples

**Figure 2:**
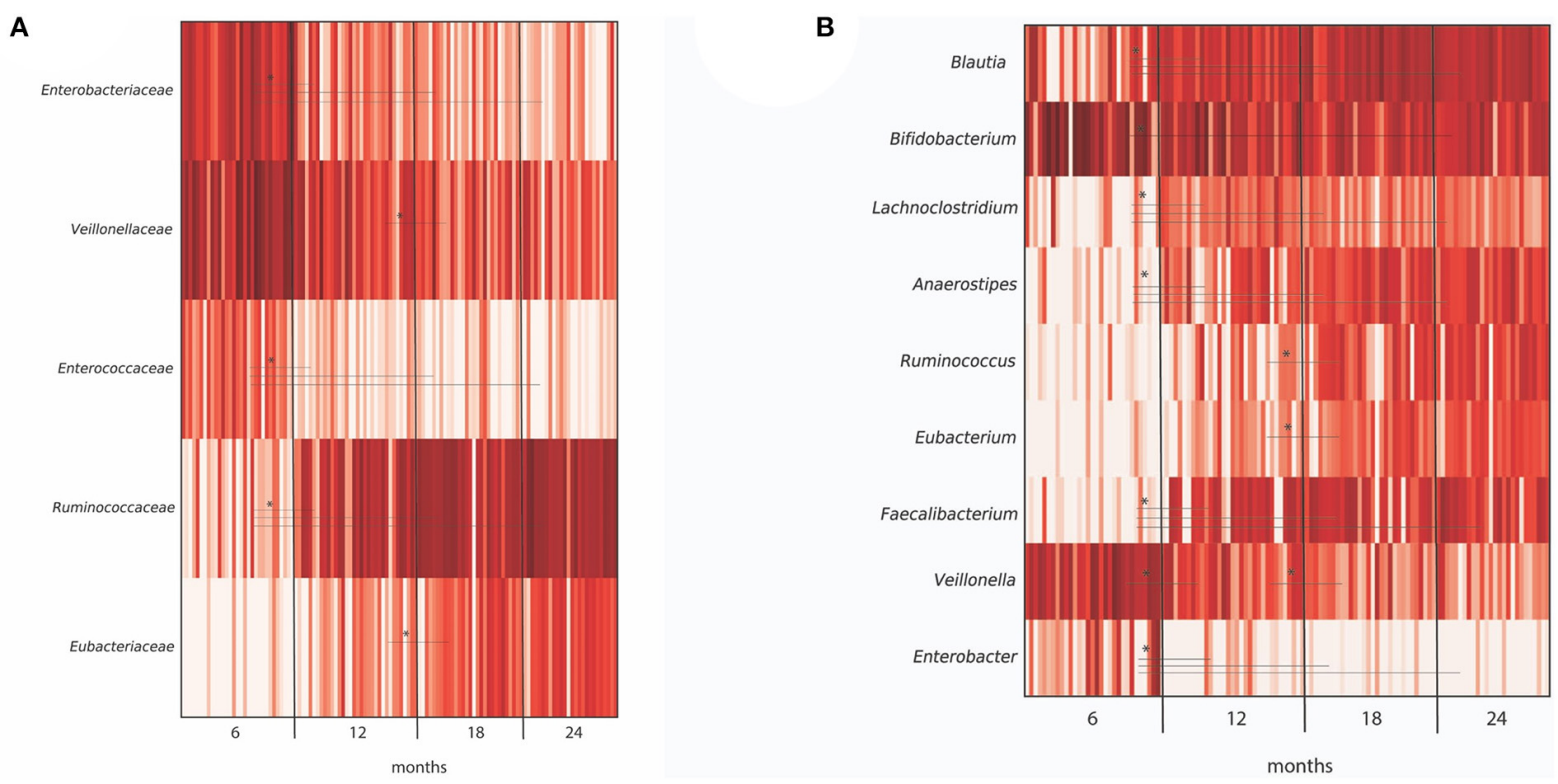
Differentially abundant taxa in microbiota from 40 infants grouped by months (6, 12, 18, and 24 months), as depicted by ANCOM analysis at **(A)** family and **(B)** genus level. For each taxa, an asterisk (*) indicates significance (*P* < 0.05) in abundance level at different time points (6, 12, 18, and 24 months). Only OTUs > 1% were included in the analysis.

Diversity analysis of infant fecal samples from Illumina sequencing data showed an increase in alpha diversity (species diversity in each sample) over the time period from 6 to 24 months (Supplementary Figure 1). In contrast, beta diversity (species dissimilarity between communities) plots from UniFrac distance matrices (weighted, unweighted, and generalized) did not show significant shifts in bacterial taxa from 6 to 24 months (Supplementary Figure 2). At the taxa level, large-individual variability was observed in relative abundance at the genus level in fecal samples of 16 infants at 6, 12, 18, and 24 months (Supplementary Figure 3). Importantly, several significant shifting of bacteria taxa were observed over time. At the family level, a significant decrease in the relative abundance of *Enterobacteriaceae* and lactate-utilizing and H_2_-producing *Veillonellaceae* were observed from 6 to 24 months (Figure 2A). In contrast, the relative abundance of BPB genera, such as *Anaerostipes, Faecalibacterium*, and H_2_-producing *Eubacterium* increased significantly from 6 to 24 months (Figure 2B).

### Analysis of functional microbial group in infant feces by cultivation and by qPCR

**Figure 3:**
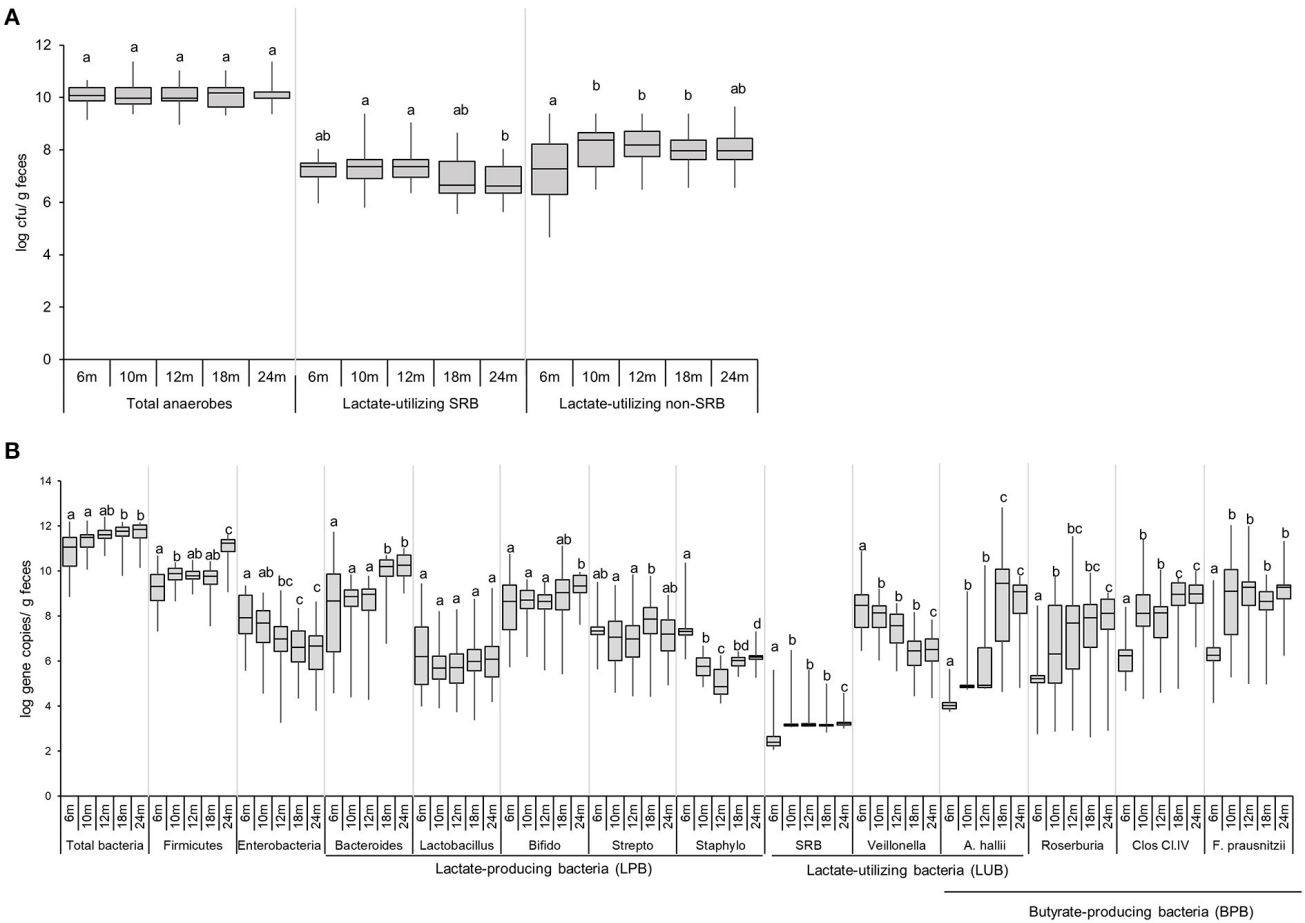
Enumeration of functional groups of microbes in infant feces (*n* = 40) using culture-based methods **(A)** and qPCR **(B)** from 6 to 24 months. Data obtained by culture-based methods are expressed as median ± IQR log cfu/g feces. Data obtained by qPCR are expressed as median ± IQR log gene copies/g feces. Data were calculated from positive samples only (Supplementary Table 3). SRB, sulfate-reducing bacteria. Significant differences between the different time points were calculated using the Kruskal Wallis test and indicated with different letters (*p* < 0.05).

The colonization of viable total anaerobes, LUB-SRB, and non-SRB, in the gut of 40 infants over time, was investigated by the most probable number cultivation method and by qPCR, and results are shown in Figure 3 and Supplementary Table 3. Total anaerobe viable counts at 6 months were high, with a median equal to 10.1 log CFU g^−1^ feces, and remained stable until 24 months (10 log CFU g^−1^) (Figure 3A). The Cul LUB SRB was detected in 97% of infants at 6 months and with prevalence ranging between 92 and 100% until 2 years of age (Supplementary Table 3). The median count of Cul LUB SRB in positive samples was maximum at 7.4 log CFU g^−1^ at 6 months and tended to decrease to 6.6 log CFU g^−1^ at 24 months. Moreover, there were significantly higher median counts of Cul LUB SRB at 10 months (7.4 log CFU g^−1^) and 12 months (7.4 log CFU g^−1^) compared to 24 months. In contrast, the numbers of viable cells of Cul LUB non-SRB groups in positive samples increased significantly from 6 months (7.3 log CFU g^−1^) to 10 months (8.4 log CFU g^−1^) and did not change until 24 months (8.0 CFU g^−1^) (Figure 3A).

The median gene copy number of total bacteria significantly increased from 11.1 log copies g^−1^ of feces at 6–18 and 24 months (11.8 and 11.9 log copies g^−1^, respectively) (Figure 3B). The level of Firmicutes, one of four dominant phyla in an infant's gut, significantly increased from 6 months (9.3 log copies g^−1^) to 10 months (9.9 log copies g^−1^), remained stable at 18 months (9.8 log copies g^−1^) and strongly increased by 1.4 logs at 24 months (11.2 log copies g^−1^). Levels of Enterobacteriaceae, a large family including many enteropathogens, such as *Salmonella, Escherichia coli, Klebsiella*, and *Shigella*, gradually decreased from 6 months (7.9 log copies g^−1^) to 12 months (7.0 log copies g^−1^), and were significantly lower at 18 and 24 months (6.6 and 6.7 log copies g^−1^) compared to 6 months. Linear regression analysis demonstrated a significant increase in levels of Firmicutes (*P* = 0.002) and a significant decrease in levels of Enterobacteriaceae (*P* = 0.046) over time (Supplementary Table 6).

Among the LPB community detected by qPCR, *Bacteroides, Lactobacillus*, and *Bifidobacterium* were present in the feces of a majority of infants during the whole investigation period (Figure 3B, Supplementary Table 3). The *Bifidobacterium* level was mainly stable from 6 months (8.7 log copies g^−1^) to 24 months (9.3 log copies g^−1^) (Figure 3B). *Bacteroides* levels remained stable from 6 months (8.7 log copies g^−1^) to 12 months (9.0 log copies g^−1^) and significantly increased at 18 and 24 months (10.2 and 10.2 log copies g^−1^, respectively) compared to the previous time points.

The LUB groups detected by qPCR comprised SRB and non-SRB LUB, *Veillonella*, and *Anaerobutyricum hallii*. SRB was detected in low prevalence over the first 2 years of life (<33%). SRB numbers in positive infants significantly increased from 6 months (2.4 log copies g^−1^) to 10 months (3.2 log copies g^−1^) and remained stable until 24 months. The prevalence and abundance of *A. hallii* steadily and sharply increased from 6 months (13% and 4.0 log copies g^−1^, respectively) until 24 months (85% and 9.1 log copies g^−1^, respectively) (Figure 3B). The propionate-producing *Veillonella* was detected in all participants from 6 to 24 months (Supplementary Table 3). *Veillonella* levels decreased steadily over time, from 8.5 log copies g^−1^ at 6 months to 6.5 log copies g^−1^ at 24 months. Linear regression analysis confirmed how *Veillonella* levels decreased while *A. hallii* levels increased over time (Supplementary Table 5).

Prevalence of BPB groups detected by qPCR, including *A. hallii, Roseburia*, Clostridium Cluster IV, and *Faecalibacterium prausnitzii*, increased over time (Figure 3B). At 6 months, the prevalence of *Roseburia*, Clostridium Cluster IV, and *F. prausnitzii* was 41%, 69%, and 54%, respectively, while they were present from 10 to 24 months in all infants (Supplementary Table 3). The prevalence of *A. hallii* also steadily increased from 6 months (13%) until 24 months (85%). We observed similar patterns between *F. prausnitzii* and Clostridium Cluster IV levels, with a significant increase by 2–3 logs observed from 6 to 10 months. *F. prausnitzii* levels were stable from 10 to 24 months, while Clostridium Cluster IV levels significantly increased from 12 to 18 months (8.1 and 9.0 log copies g^−1^) and were stable from 18 to 24 months (9.0 log copies g^−1^) (Figure 3B).

### Metabolite profiles in infant feces

**Figure 4:**
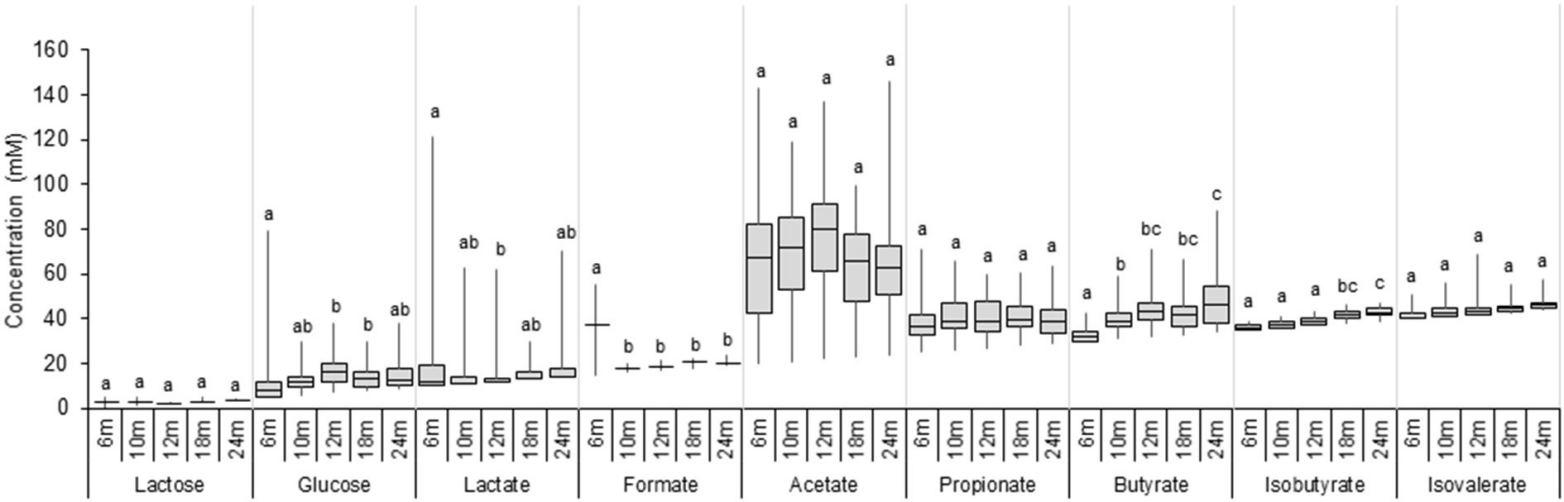
Metabolite concentrations in fecal samples of infants from 6 to 24 months (*n* = 40). Values are expressed as median ± IQR. Significant differences between the different time points were calculated using the Kruskal Wallis test and indicated with different letters (*p* < 0.05).

Lactose, glucose, lactate, SCFA, and BCFA were measured in extracted fecal water using HPLC, and the results are shown in Figure 4 and Supplementary Table 4. Large inter-individual variability in metabolic profiles over time was observed in our cohort (Supplementary Figure 4). Lactose was detected in a few participants (3–8%) at very low levels (<5 mM). In contrast, glucose was found in a fecal sample of a majority of infants from 6 to 24 months (increasing from 65 to 100%; Supplementary Table 4), and fecal glucose concentration showed high variations with an increase from 6 (2.9 mM) to 12 months (9.0 mM) (*P* < 0.05, Figure 4). The intermediate metabolites, lactate, and formate were frequently detected in fecal samples. The prevalence of lactate gradually decreased over time from 60% at 6 months to 35% at 24 months, and the median fecal lactate concentration significantly decreased from 6 months (2.0 mM) to 12 months (0.0 mM) and remained unchanged until 24 months. Formate was detected in 20% of the infants at 6 months and in only one infant feces from 10 to 24 months (3 %). Acetate and propionate were present in a majority of infants during the whole investigation period with high individual variations (Supplementary Table 4). Acetate was the major detected SCFA, with median levels remaining stable between 6 and 24 months (59.5–57.2 mM). Similarly, fecal propionate detected in a majority of infant feces (92–100% prevalence) exhibited stable levels from 6 until 24 months (11.1–14.2 mM). An increase in the prevalence of fecal butyrate from 73% at 6 months to 100% at 24 months was observed. Butyrate was detected in low amounts at 6 months (2.1 mM), and the concentration steadily increased over time to 15.6 mM at 24 months as revealed by linear regression analysis (*P* < 0.05; Supplementary Table 5). BCFA Isobutyrate and isovalerate were detected in approximately half of the infants at 6 months, and in 98 and 78% at 24 months, respectively (Supplementary Table 4). BCFA concentration remained low (median <4 mM) in infant fecal samples from 6 to 24 months. A significant increase in mean isobutyrate concentration was observed from 12 to 18 months (1.7 and 3.7 mM) (Figure 4).

### Correlation analyses between functional groups and metabolites in infant feces

**Figure 5:**
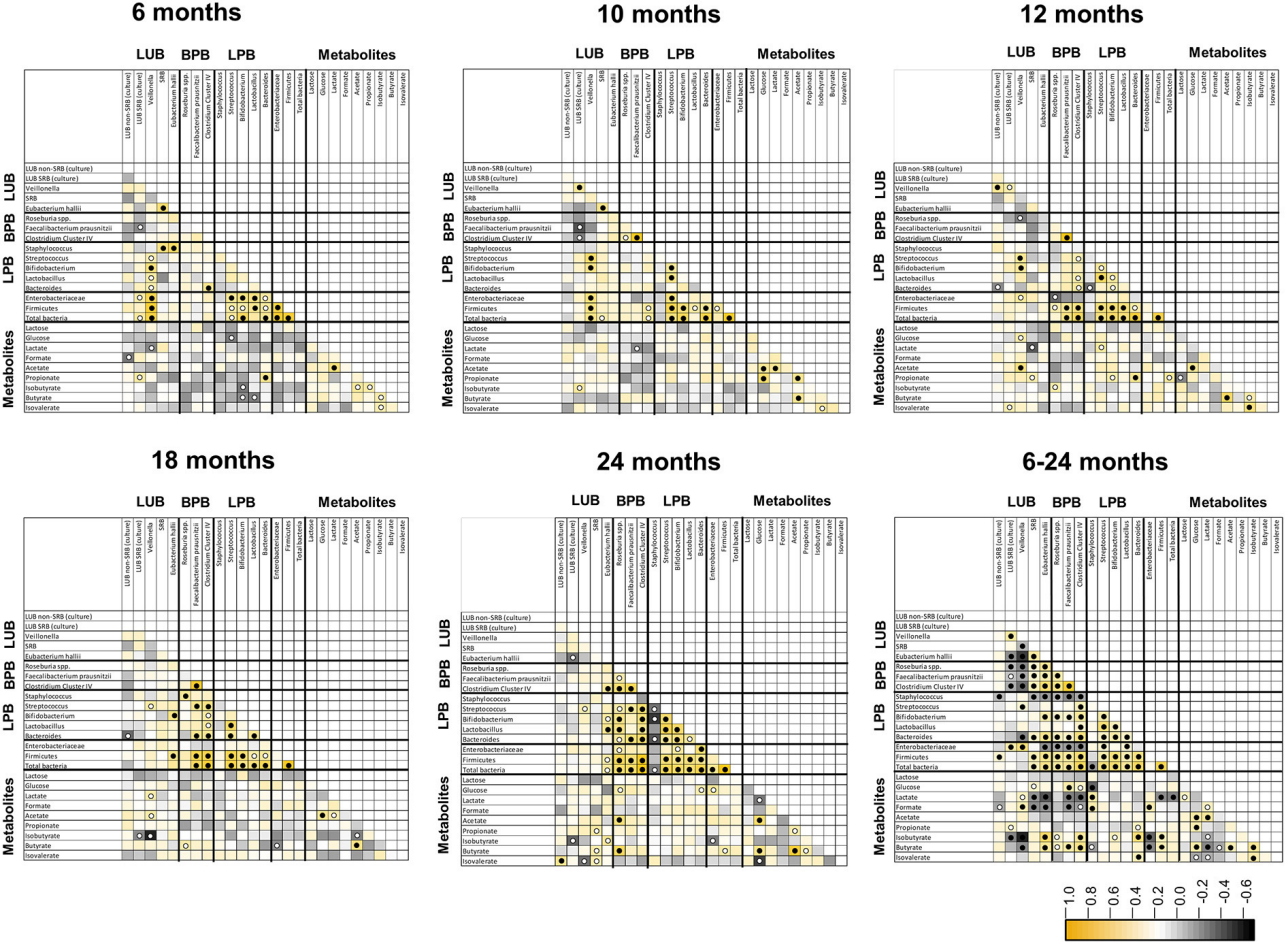
Spearman pairwise correlation map of measured bacterial 16S rRNA gene targets and fecal metabolite concentrations from 6 to 24 months of life (*n* = 40). The color gradient denotes the Spearman *R*-value. White circle, *q* < 0.05; black circle, *q* < 0.01. LUB, lactate-utilizing bacteria; SRB, sulfate-reducing bacteria.

Spearman's correlations between functional groups (LPB, LUB, and BPB) and metabolites SCFA and BCFA at five time points in infant feces from 6 to 24 months are depicted in Figure 5. Significant positive correlations were observed between LUB and LPB and for H_2_-producing and H_2_-utilizing taxa at different timepoints. LUB *Veillonella* correlated positively with LPB *Streptococcus* at 6, 10, 12, 18, and 24 months, with *Bifidobacterium* at 6, 10, and 12 months, and with *Lactobacillus* at 6 months. Significant positive correlations were also observed between LUB *A. hallii* and LPB *Bifidobacterium* at 18 and 24 months. H_2_-producing *A. hallii* and *Veillonella* correlated positively with H_2_-utilizing SRB at 6 and 10 months and Cul LUB SRB at 10 and 12 months, respectively.

Correlation analysis further revealed positive correlations between metabolites. Acetate correlated positively with lactate from 6 months to 10 months, glucose at 10, 12, 18, and 24 months, propionate at 10 months of life, and butyrate from 10 to 24 months.

Correlation analysis of pooled data from 6 to 24 months also confirmed timepoint comparisons and revealed additional significant correlations between LUB and BPB. Over the 1.5-years, LUB SRB (culture) and *Veillonella* negatively correlated with BPB *Roseburia* spp., while *F. prausnitzii* and Clostridium Cluster IV positively correlated with each other. Moreover, SRB and *A. hallii* positively correlated with the abovementioned BPB. Among the LUB community, *Veillonella* and *A. hallii* positively correlated with LUB SRB (culture) and SRB, respectively. In contrast, *Veillonella* correlated negatively with SRB and *A. hallii*. Most of the LPBs correlated positively with each other. *Bifidobacterium* and *Bacteroides* correlated positively with all BPB.

Lactate correlated positively with LUB *Veillonella* and with *Staphylococcus*, and negatively with SRB, *A. hallii, F. prausnitzii*, Clostridium Cluster IV, Firmicutes, and total bacteria. Propionate correlated positively with *Bacteroides*-producing propionate and with LUB SRB (culture). As expected, butyrate correlated positively with all BPB. Furthermore, butyrate correlated negatively with *Veillonella, Staphylococcus*, and Enterobacteriaceae. Among the metabolites, lactate positively correlated with lactose, formate, and acetate, and negatively correlated with butyrate, isobutyrate, and isovalerate (Figure 5).

### Impact of delivery mode and infant colics on the colonization of functional microbial groups

**Figure 6:**
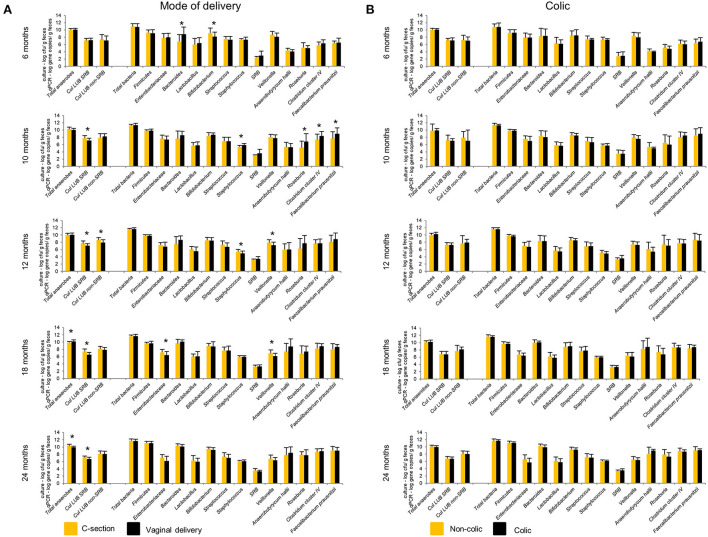
Effect of mode of delivery **(A)** and colic **(B)** on infant gut microbiota at 6, 10, 12, 18, and 24 months. Values obtained by culture-based methods are expressed as means ± SD log cfu/g feces. Values obtained by qPCR are expressed as means ± SD log gene copies/g feces. Means were compared pairwise using Student's t-test for normally distributed data. Non-parametric Mann–Whitney test was performed when data were not normally distributed. *P* < 0.05 were considered significant. ^*^P < 0.05. LUB, lactate-utilizing bacteria; SRB, sulfate-reducing bacteria.

Our previous study showed significantly lower levels of *Bacteroides* detected by qPCR in infants delivered by CS (*n* = 11) compared to VD (*n* = 29) infants from 2 weeks to 6 months (15). This difference disappeared from 10 to 24 months (Figure 6). At 10 months, CS infants harbored significantly lower numbers of BPB, including *Roseburia*, Clostridium cluster IV, and *F. prausnitzii* compared to VD infants. Infants delivered by CS had significantly higher Cul LUB SRB levels compared to VD infants at 10, 12, 18, and 24 months. Furthermore, lactate-utilizing *Veillonella* number was higher in CS infants at 12 and 18 months. LDA effect size (LEfSe) algorithm used to identify taxa with differing abundance in samples from VD and CS infants showed that VD infants had fecal samples enriched in *Collinsella* at 6 months (Supplementary Figure 5). At 12 and 18 months, fecal samples of VD infants were enriched in the *Veillonella* genus and Veillonellaceae family.

No significant differences in the gut microbiota composition and metabolite concentrations were found between colicky (*n* = 8) and non-colicky (*n* = 32) infant groups from 6 to 24 months, except for isobutyrate and isovalerate (Figure 6, Supplementary Table 6). LEfSe analysis of sequencing data showed that colicky infants had fecal samples enriched in *Ruminococcus* at 6 months compared to non-colicky infants (Supplementary Figure 6). At 12 months, fecal samples of non-colic infants were enriched in the *Eubacterium* genus and Eubacteriaceae family. Colicky infants had fecal samples enriched in *Anaerostipes* and Coriobacteriaceae family at 18 and 24 months, respectively.

## Discussion

Early intestinal colonization plays an essential role in infant gut health. The period of 6–12 months corresponds to a transition in the infant diet, from mostly breast milk or infant formula milk-based to a mix of nutritionally adequate solid foods and liquids (31). Only a few studies have investigated the changes occurring in the gut microbiota composition and metabolism during this “critical window” for establishing long-term dietary practices and future health outcomes (32). We present here a longitudinal study with seven timepoints that cover the period from 6 to 24 months of life, with focus on the colonization of important microbial functional groups involved in lactate metabolism and butyrate production using, for the first time, a combination of traditional and molecular methods. Our data complement the first 6 months colonization study previously reported by Pham et al. (15). Taking all data together, we monitored the natural development of the infant gut microbiota in 40 healthy infants from 0 to 24 months, vaginally-delivered (*n* = 29) or cesarian-born (*n* = 11), and non-colicky (*n* = 32), or colicky (*n* = 8) infants. We observed a convergence to a more uniform composition and metabolic activity upon the 24 months initial maturation.

### A shifted colonization pattern in an infant's gut over time with a succession of functional groups

Consistent with previous findings, we observed changes in levels of dominant microbial groups, where levels of Firmicutes increased (*P* < 0.05) and *Enterobacteriaceae* decreased (*P* < 0.05) over 24 months (10, 33). We have previously seen a decrease of LPB from 2 weeks to 6 months, mainly *Bifidobacterium, Streptococcus*, and *Staphyloccocus* (15), which extended until 24 months in this study. The decrease in *Bifidobacterium* level and abundance could be partially explained by gut maturation and reduced consumption of breast milk containing HMOs, which are well-known for bifidogenic effects (13, 34). Recently, the key ecological role of *Bifidobacterium* in providing substrates for other important colonizers such as *Anaerostipes caccae* in the infant's gut has been highlighted (18).

In this study, we observed an increase in levels of LPB at 18 and 24 months, including *Bacteroides*. Members of *Bacteroides* were shown to possess wide saccharolytic potential, with the ability to degrade complex glycans (35). Hence, the predominance of *Bacteroides* could be explained by the impact of dietary transition from breast/formula milk to solid foods that contain more fibers.

In our study, correlations between LPB and LUB over the first 2 years of life support the metabolic cross-feeding of lactate in the infant's gut. We previously observed an accumulation of lactate in feces during the first 6 months of life, however, with high inter-individual variability (15). Lactate accumulation in fecal samples reflects the balance between production, utilization, and intestinal absorption. At 10 months of age, lactate was only detected in a minority of infants, with a concentration comparable to adult feces (36). Another main finding is the switch in the predomination of LUB during the first 2 years of life, where *Veillonella* and *A. hallii* dominated the niche during the first and second year of life, respectively. The predominance of LUB *Veillonella* over the first year has been recently shown in a longitudinal study of 471 children from birth to 5 years (37). While *Veillonella* cannot ferment carbohydrates and rely solely on L-lactate as the main energy-yielding carbon substrate to form propionate and H_2_, *A. hallii* is capable of metabolizing glucose and fermentation intermediates (i.e. acetate and both lactate isomers) to form butyrate and H_2_. Moreover, the ability of *A. hallii* to convert 1,2-propanediol to propionate was previously reported (38, 39). Therefore, compared to other LUB, the ability of *A. hallii* to metabolize a wide range of substrates might promote colonization and proliferation in the infant's gut after the introduction of complementary foods. In our cohort, formate was detected in a small fraction of the population at low levels. Formate can be produced by *A. hallii* and by bifidobacteria, and utilized by methanogens or acetogens, which are usually not detected in infants (40, 41).

While Cul LUB non-SRB levels increased over 24 months, SRB levels detected by both cultural and molecular methods remained unchanged over time. Especially, the high prevalence of viable SRB (>90%) from 6 to 24 months with levels comparable to those found in adult and healthy children suggests a role as a consistent component of the core infant gut microbiota (42–44). The high prevalence and levels of Cul LUB SRB might be explained by metabolic cross-feeding of H_2_, where H_2_ produced by both *Veillonella* and *A. hallii* serve as a preferred substrate for SRB (45, 46). Consistent with this notion, we report significant positive correlations between SRB and *A. hallii* at 6, 10 months, and across the 6–24 months study; and between Cul LUB SRB and *Veillonella* at 10 and 12 months. The balance between H_2_ producers and utilizers might be crucial to infant gut health because increased H_2_ production by LUB might result in gas accumulation and bloating generating. On the other hand, the early and consistent presence of SRB may be of clinical significance for the infant's gut because SRB reduces sulfate to H_2_S, which can be toxic for colonic epithelial cells and inhibit butyrate oxidation in the cells (47).

From 6 months to 2 years of life, acetate was detected in fecal samples of all infants and remained the major SCFA, albeit inter-individual variability was considerable. Propionate was detected in the majority of the infants from 6 to 24 months. All SCFA, including isoacids, showed increased concentrations within the first 2 years, with specific patterns. Acetate correlated positively with propionate in the first 10 months of life and with butyrate from 10 to 24 months, which is consistent with an increase of BPB that can produce butyrate from net acetate.

Butyrate is the major energy source for colonocytes and has been implicated in the prevention of inflammatory bowel diseases (48, 49). Surprisingly, little is known about the colonization pattern of functional groups producing butyrate in the infant's gut. Our study detected different BPB taxa, with *Roseburia*, Clostridium cluster IV, and *F. prausnitzii* detected in significant numbers in all 10 and 12 months infant feces, whereas *A. hallii* was detected in only 25 and 40% of infants, respectively. The observed increase of BPB *A. hallii, F. prausnitzii*, and butyrate-producing taxa Clostridium cluster IV were reported in two infant cohort studies (10, 37), whereas *Faecalibacterium* was also detected at 12 months in a recent cohort with 229 infants (50). The role of *A. hallii* as key species utilizing intermediates of HMO fermentation by bifidobacteria was previously reported (41).

BPB, including *Butyrivibrio crossotus, Eubacterium rectale*, and *A. hallii*, were negatively associated with crying and were more abundant in healthy infants than colicky infants (51). Concomitantly, our study showed that fecal samples of non-colicky infants were enriched in *Eubacterium* at 12 months. Interestingly, we observed significantly lower numbers of BPB, including *Roseburia*, Clostridium cluster IV, and *F. prausnitzii* in CS compared to VD infants at 10 months, suggesting that the mode of delivery might have an impact on the colonization pattern of beneficial microbes such as BPB.

Analysis of functional genes involved in butyrate production showed that there is still a fraction of BPB strains that have not yet been cultured. *Anaerostipes rhamnosivorans* sp. nov., a human intestinal, butyrate-producing bacterium, was isolated from a stool sample of a 1-year infant (52). In the future, similar efforts should be devoted to isolating novel BPB during the first years of life. Furthermore, the prevalence and levels of other BPB that were isolated from adult fecal samples, such as *A. caccae, A. coli, E. rectale*, and *E. limosum*, as well as functional genes involved in butyrate production pathways such as butyryl-CoA: acetate CoA transferase (encoded by *but*) or butyrate kinase (encoded by *buk*; after phosphorylation of butyryl-CoA), are limited and should be investigated in further studies (53).

Interestingly, the specific LUB signatures for colicky infants observed in the first 6 months of life disappeared from 6 months to 2 years old, suggesting that the imbalance in LUB colonization, which contributes to colicky symptoms, is not persistent, but limited to the first few months of life.

We acknowledge several limitations and outlooks of this study. From a dietary perspective, an infant's gut microbial profile is highly individual, unstable, and influenced by a number of factors over the first year of life. Our study included a limited number of infants who were all recruited in the Zürich region. Furthermore, we could not collect accurate dietary information. Because dietary habits are influenced by many factors, including geography and culture, and because complementary feeding practices vary considerably across low-, middle-, and high-income countries, investigating large infant cohorts in different regions would be granted to support the development of guidelines surrounding the critical timing of the introduction to complementary foods. The inclusion of nutritional surveys will allow further investigation of the impact of infant diet on microbiota composition and function. Lastly, we did not investigate the virome and mycobiome of this infant cohort. Given that fungi and viruses may be involved in the modulation of infant health, future studies, including the bacteriome, virome, and mycobiome, and their complex interactions, are needed.

## Conclusion

In conclusion, our longitudinal study of 40 Swiss infants provides important insights into the colonization of functional groups involved in lactate metabolism and butyrate production during the critical development period from 6 months to 2 years. Our data from a combination of cultivation and molecular methods captured the dynamic changes of the infant gut microbiota and informed on the switch of predominant LPB, from *Bifidobacterium* in the first year to *Bacteroides* in the second year of life, and the predominant LUB, from *Veillonella* to *A. hallii*. The significant increase in BPB and fecal butyrate concentration was also pinpointed to the period between 6 and 10 months, corresponding to the weaning period. Our correlation analyses further demonstrated the metabolic cross-feeding of lactate, and for the first time, of H_2_. The healthy infant gut microbiota composition and metabolic activity in the first 2 years of life are highly flexible, and hence provide the potential for stirring its development *via* dietary factors, including the introduction of solid foods and the development of pro- and prebiotics for modulation of the gut microbiota toward long-lasting health.

## Data availability statement

The data presented in the study are deposited in the ENA repository, accession number PRJEB53435, https://www.ebi.ac.uk/ena/browser/view/PRJEB53435?show=reads.

## Ethics statement

The studies involving human participants were reviewed and approved by Ethic Committee of ETH Zurich (Project EK 2012-N-36; date of approval 28.09.2012). Written informed consent to participate in this study was provided by the participants' legal guardian/next of kin.

## Author contributions

VP, CC, CB, and CL conceptualized and managed the study. VP and AG contributed to sample collection, data generation, analyzed the data, and drafted the manuscript. VP, AG, and CL reviewed and edited the manuscript. All authors contributed to the article and approved the submitted version.

## Funding

Financial support for this work was provided by the Swiss National Science Foundation (project number: 310030_146784, Bern, Switzerland). Open access funding was provided by ETH Zurich.

## Conflict of interest

The authors declare that the research was conducted in the absence of any commercial or financial relationships that could be construed as a potential conflict of interest.

## Publisher's note

All claims expressed in this article are solely those of the authors and do not necessarily represent those of their affiliated organizations, or those of the publisher, the editors and the reviewers. Any product that may be evaluated in this article, or claim that may be made by its manufacturer, is not guaranteed or endorsed by the publisher.

## References

[1] Wopereis H Oozeer R Knipping K Belzer C Knol J. The first thousand days - intestinal microbiology of early life: establishing a symbiosis. Pediatr Allergy Immunol. (2014) 25:428–38. 10.1111/pai.12232 24899389

[2] Dunn AB Jordan S Baker BJ Carlson NS. The maternal infant microbiome: considerations for labor and birth. Am J Matern Child Nurs. (2017) 42:318–25. 10.1097/NMC.0000000000000373 28825919 PMC5648605

[3] Ferretti P Pasolli E Tett A Asnicar F Gorfer V Fedi S. Mother-to-Infant microbial transmission from different body sites shapes the developing infant gut microbiome. Cell Host Microbe. (2018) 24:133–45.e5. 10.1016/j.chom.2018.06.005 30001516 PMC6716579

[4] Korpela K Costea P Coelho LP Kandels-Lewis S Willemsen G Boomsma DI. Selective maternal seeding and environment shape the human gut microbiome. Genome Res. (2018) 28:561–8. 10.1101/gr.233940.117 29496731 PMC5880245

[5] Zhuang L Chen H Zhang S Zhuang J Li Q Feng Z. Intestinal microbiota in early life and its implications on childhood health. Genomics Proteomics Bioinformatics. (2019) 17:13–25. 10.1016/j.gpb.2018.10.002 30986482 PMC6522475

[6] Walker WA. The importance of appropriate initial bacterial colonization of the intestine in newborn, child, adult health. Pediatr Res. (2017) 82:387–95. 10.1038/pr.2017.111 28426649 PMC5570628

[7] Jost T Lacroix C Braegger CP Chassard C. New insights in gut microbiota establishment in healthy breast fed neonates. PLoS ONE. (2012) 7:e44595. 10.1371/journal.pone.0044595 22957008 PMC3431319

[8] Avershina E Lundgard K Sekelja M Dotterud C Storro O Oien T. Transition from infant- to adult-like gut microbiota. Environ Microbiol. (2016) 18:2226–36. 10.1111/1462-2920.13248 26913851

[9] Koenig JE Spor A Scalfone N Fricker AD Stombaugh J Knight R. Succession of microbial consortia in the developing infant gut microbiome. Proc Natl Acad Sci USA. (2011) 108 (Suppl. 1):4578–85. 10.1073/pnas.1000081107 20668239 PMC3063592

[10] Bergstrom A Skov TH Bahl MI Roager HM Christensen LB Ejlerskov KT. Establishment of intestinal microbiota during early life: a longitudinal, explorative study of a large cohort of Danish infants. Appl Environ Microbiol. (2014) 80:2889–900. 10.1128/AEM.00342-14 24584251 PMC3993305

[11] Backhed F Roswall J Peng Y Feng Q Jia H Kovatcheva-Datchary P. Dynamics and stabilization of the human gut microbiome during the first year of life. Cell Host Microbe. (2015) 17:690–703. 10.1016/j.chom.2015.04.004 25974306

[12] Bokulich NA Chung J Battaglia T Henderson N Jay M Li H. Antibiotics, birth mode, and diet shape microbiome maturation during early life. Sci Transl Med. (2016) 8:343ra382. 10.1126/scitranslmed.aad7121 27306664 PMC5308924

[13] de Muinck EJ Trosvik P. Individuality and convergence of the infant gut microbiota during the first year of life. Nat Commun. (2018) 9:2233. 10.1038/s41467-018-04641-7 29884786 PMC5993781

[14] Chassard C Lacroix C. Carbohydrates and the human gut microbiota. Curr Opin Clin Nutr Metab Care. (2013) 16:453–60. 10.1097/MCO.0b013e3283619e63 23719143

[15] Pham VT Lacroix C Braegger CP Chassard C. Early colonization of functional groups of microbes in the infant gut. Environ Microbiol. (2016) 18:2246–58. 10.1111/1462-2920.13316 27059115

[16] Bunesova V Lacroix C Schwab C. Mucin cross-feeding of infant bifidobacteria and *Eubacterium hallii*. Microb Ecol. (2018) 75:228–38. 10.1007/s00248-017-1037-4 28721502

[17] Rocha Martin VN Schwab C Krych L Voney E Geirnaert A Braegger C. Colonization of *Cutibacterium avidum* during infant gut microbiota establishment. FEMS Microbiol Ecol. (2019) 95:fiy215. 10.1093/femsec/fiy215 30388209

[18] Chia LW Mank M Blijenberg B Bongers RS van Limpt K Wopereis H. Cross-feeding between *Bifidobacterium infantis* and *Anaerostipes caccae* on lactose and human milk oligosaccharides. Benef Microbes. (2021) 12:69–83. 10.3920/BM2020.0005 33191780

[19] Dobbler P Mai V Procianoy RS Silveira RC Corso AL Roesch LFW. The vaginal microbial communities of healthy expectant Brazilian mothers and its correlation with the newborn's gut colonization. World J Microbiol Biotechnol. (2019) 35:159. 10.1007/s11274-019-2737-3 31602538 PMC6787113

[20] Ewaschuk JB Naylor JM Zello GA. D-lactate in human and ruminant metabolism. J Nutr. (2005) 135:1619–25. 10.1093/jn/135.7.1619 15987839

[21] Monroe GR van Eerde AM Tessadori F Duran KJ Savelberg SMC van Alfen JC. Identification of human D lactate dehydrogenase deficiency. Nat Commun. (2019) 10:1477. 10.1038/s41467-019-09458-6 30931947 PMC6443703

[22] Pham VT Lacroix C Braegger CP Chassard C. Lactate-utilizing community is associated with gut microbiota dysbiosis in colicky infants. Sci Rep. (2017) 7:11176. 10.1038/s41598-017-11509-1 28894218 PMC5593888

[23] Pham VT Chassard C Rifa E Braegger C Geirnaert A Rocha Martin VN. Lactate metabolism is strongly modulated by fecal inoculum, pH, and retention time in polyferms continuous colonic fermentation models mimicking young infant proximal colon. mSystems. (2019) 4:e00264–18. 10.1128/mSystems.00264-18 31138674 PMC6538849

[24] Duncan SH Barcenilla A Stewart CS Pryde SE Flint HJ. Acetate utilization and butyryl coenzyme A (CoA):acetate-CoA transferase in butyrate-producing bacteria from the human large intestine. Appl Environ Microbiol. (2002) 68:5186–90. 10.1128/AEM.68.10.5186-5190.2002 12324374 PMC126392

[25] Canani RB Costanzo MD Leone L Pedata M Meli R Calignano A. Potential beneficial effects of butyrate in intestinal and extraintestinal diseases. World J Gastroenterol. (2011) 17:1519–28. 10.3748/wjg.v17.i12.1519 21472114 PMC3070119

[26] Chen J Vitetta L. Inflammation-Modulating effect of butyrate in the prevention of colon cancer by dietary fiber. Clin Colorectal Cancer. (2018) 17:e541–4. 10.1016/j.clcc.2018.05.001 29866614

[27] Silva JPB Navegantes-Lima KC Oliveira ALB Rodrigues DVS Gaspar SLF Monteiro VVS. Protective mechanisms of butyrate on inflammatory bowel disease. Curr Pharm Des. (2018) 24:4154–66. 10.2174/1381612824666181001153605 30277149

[28] Oguntoyinbo FA Cho GS Trierweiler B Kabisch J Rosch N Neve H. Fermentation of African kale (*Brassica carinata*) using *L. plantarum* BFE 5092 and *L. fermentum* BFE 6620 starter strains. Int J Food Microbiol. (2016) 238:103–12. 10.1016/j.ijfoodmicro.2016.08.030 27614122

[29] Hungate R. A roll tube method for the cultivation of strict anaerobes. In: Norris JR Ribbons DW, editors. Method Microbiology 3B. (1969). p. 117–32. 10.1016/S0580-9517(08)70503-8

[30] Mandal S Van Treuren W White RA Eggesbo M Knight R Peddada SD. Analysis of composition of microbiomes: a novel method for studying microbial composition. Microb Ecol Health Dis. (2015) 26:27663. 10.3402/mehd.v26.27663 26028277 PMC4450248

[31] World Medical A. World medical association declaration of Helsinki. Ethical principles for medical research involving human subjects. Bull World Health Organ. (2001) 79:373–4. 10.4414/fms.2001.04031 11357217 PMC2566407

[32] Rippey PLF Aravena F Nyonator JP. Health impacts of early complementary food introduction between formula-fed and breastfed infants. J Pediatr Gastroenterol Nutr. (2020) 70:375–80. 10.1097/MPG.0000000000002581 31834112

[33] Pannaraj PS Li F Cerini C Bender JM Yang S Rollie A. Association between breast milk bacterial communities and establishment and development of the infant gut microbiome. JAMA Pediatr. (2017) 171:647–54. 10.1001/jamapediatrics.2017.0378 28492938 PMC5710346

[34] Walker WA Iyengar RS. Breast milk, microbiota, and intestinal immune homeostasis. Pediatr Res. (2015) 77:220–8. 10.1038/pr.2014.160 25310762

[35] Flint HJ Scott KP Duncan SH Louis P Forano E. Microbial degradation of complex carbohydrates in the gut. Gut Microbes. (2012) 3:289–306. 10.4161/gmic.19897 22572875 PMC3463488

[36] Mayeur C Gratadoux JJ Bridonneau C Chegdani F Larroque B Kapel N. Faecal D/L lactate ratio is a metabolic signature of microbiota imbalance in patients with short bowel syndrome. PLoS ONE. (2013) 8:e54335. 10.1371/journal.pone.0054335 23372709 PMC3553129

[37] Roswall J Olsson LM Kovatcheva-Datchary P Nilsson S Tremaroli V Simon MC. Developmental trajectory of the healthy human gut microbiota during the first 5 years of life. Cell Host Microbe. (2021) 29:765–76.e3. 10.1016/j.chom.2021.02.021 33794185

[38] Engels C Ruscheweyh HJ Beerenwinkel N Lacroix C Schwab C. the common gut microbe *Eubacterium hallii* also contributes to intestinal propionate formation. Front Microbiol. (2016) 7:713. 10.3389/fmicb.2016.00713 27242734 PMC4871866

[39] Louis P Flint HJ. Formation of propionate and butyrate by the human colonic microbiota. Environ Microbiol. (2017) 19:29–41. 10.1111/1462-2920.13589 27928878

[40] Vanderhaeghen S Lacroix C Schwab C. Methanogen communities in stools of humans of different age and health status and co-occurrence with bacteria. FEMS Microbiol Lett. (2015) 362:fnv092. 10.1093/femsle/fnv092 26025070

[41] Schwab C Ruscheweyh HJ Bunesova V Pham V Beerenwinkel N Lacroix C. Trophic interactions of infant bifidobacteria and *Eubacterium hallii* during L-fucose and fucosyllactose degradation. Front Microbiol. (2016) 8:95. 10.3389/fmicb.2017.00095 28194144 PMC5277004

[42] Fite A Macfarlane GT Cummings JH Hopkins MJ Kong SC Furrie E. Identification and quantitation of mucosal and faecal desulfovibrios using real time polymerase chain reaction. Gut. (2004) 53:523–9. 10.1136/gut.2003.031245 15016746 PMC1774019

[43] Collado MC Calabuig M Sanz Y. Differences between the fecal microbiota of coeliac infants and healthy controls. Curr Issues Intest Microbiol. (2007) 8:9–14 17489434

[44] Chassard C Dapoigny M Scott KP Crouzet L Del'homme C Marquet P. Functional dysbiosis within the gut microbiota of patients with constipated-irritable bowel syndrome. Aliment Pharmacol Ther. (2012) 35:828–38. 10.1111/j.1365-2036.2012.05007.x 22315951

[45] Duncan SH Louis P Flint HJ. Lactate-utilizing bacteria, isolated from human feces, that produce butyrate as a major fermentation product. Appl Environ Microbiol. (2004) 70:5810–7. 10.1128/AEM.70.10.5810-5817.2004 15466518 PMC522113

[46] Rey FE Gonzalez MD Cheng J Wu M Ahern PP Gordon JI. Metabolic niche of a prominent sulfate-reducing human gut bacterium. Proc Natl Acad Sci USA. (2013) 110:13582–7. 10.1073/pnas.1312524110 23898195 PMC3746858

[47] Roediger WE Duncan A Kapaniris O Millard S. Reducing sulfur compounds of the colon impair colonocyte nutrition: implications for ulcerative colitis. Gastroenterology. (1993) 104:802–9. 10.1016/0016-5085(93)91016-B 8440437

[48] Pryde SE Duncan SH Hold GL Stewart CS Flint HJ. The microbiology of butyrate formation in the human colon. FEMS Microbiol Lett. (2002) 217:133–9. 10.1111/j.1574-6968.2002.tb11467.x 12480096

[49] Gill PA van Zelm MC Muir JG Gibson PR. Review article: short chain fatty acids as potential therapeutic agents in human gastrointestinal and inflammatory disorders. Aliment Pharmacol Ther. (2018) 48:15–34. 10.1093/oso/9780198795339.003.0005 29722430

[50] Coker MO Laue HE Hoen AG Hilliard M Dade E Li Z. Infant feeding alters the longitudinal impact of birth mode on the development of the gut microbiota in the first year of life. Front Microbiol. (2021) 12:642197. 10.3389/fmicb.2021.642197 33897650 PMC8059768

[51] de Weerth C Fuentes S Puylaert P de Vos MW. Intestinal microbiota of infants with colic: development and specific signatures. Pediatrics. (2013) 131:e550–8. 10.1542/peds.2012-1449 23319531

[52] Bui TP de Vos WM Plugge CM. *Anaerostipes rhamnosivorans* sp. nov., a human intestinal, butyrate-forming bacterium. Int J Syst Evol Microbiol. (2014) 64 (Pt. 3):787–93. 10.1099/ijs.0.055061-0 24215821

[53] Vital M Howe AC Tiedje JM. Revealing the bacterial butyrate synthesis pathways by analyzing (meta)genomic data. MBio. (2014) 5:e00889. 10.1128/mBio.00889-14 24757212 PMC3994512

