# Erratum: Stepwise establishment of functional microbial groups in the infant gut between 6 months and 2 years: A prospective cohort study

**DOI:** 10.3389/fnut.2023.1181477

**Published:** 2023-03-21

**Authors:** Frontiers Production Office

**Affiliations:** Frontiers Media SA, Lausanne, Switzerland

**Keywords:** infant gut microbiota, lactate utilization, colonization, functional community, butyrate-producing bacteria

An omission to the funding section of the original article was made in error. The following sentence has been added: “Open access funding was provided by ETH Zurich.”

The original article has been updated.

