# Supplementary material for: Stepwise establishment of functional microbial groups in the infant gut between 6 months and 2 years: A prospective cohort study
Source: Front Nutr. 2022 Jul 28;9:948131. doi: 10.3389/fnut.2022.948131 (PMC9366138; doi:10.3389/fnut.2022.948131)
Supplement: Supplementary file 1 [file Data_Sheet_1.DOCX]

**Figure S1**. Change in alpha-diversities of gut microbiota (n = 40) at 4 timepoints expressed as number of observed species.


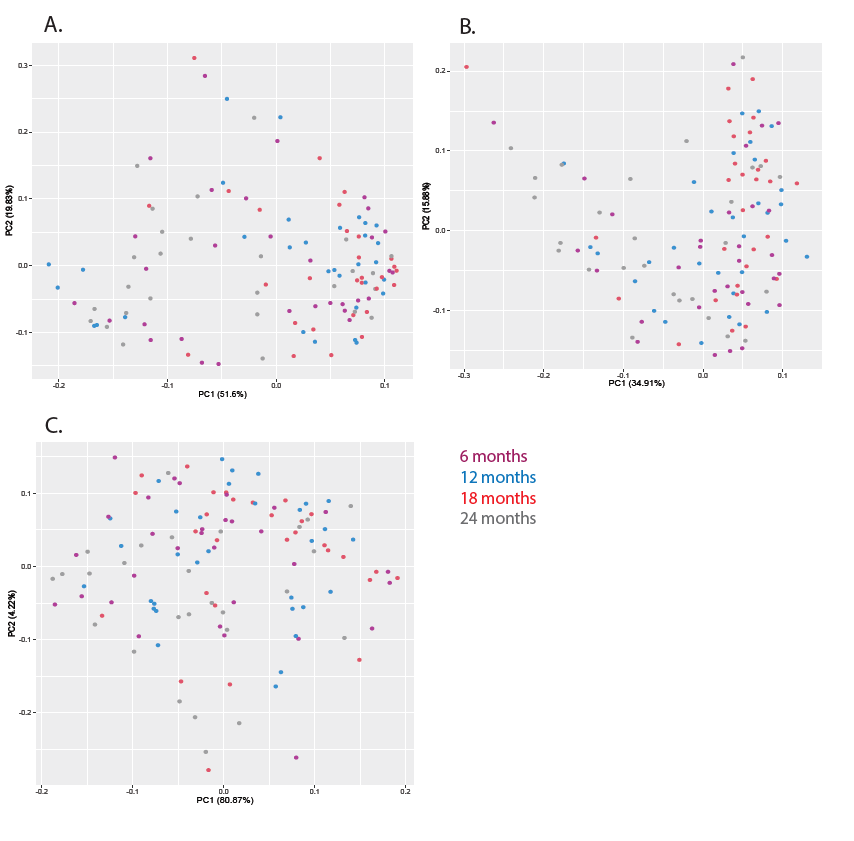


**Figure S2**. Beta diversity PCA plots constructed from weighted (A.), unweighted (B.) and generalized (C.) UniFrac distance matrix of gut microbiota from 40 infants along time: 6 months (purple), 12 months (blue), 18 months (red), 24 months (grey).

**
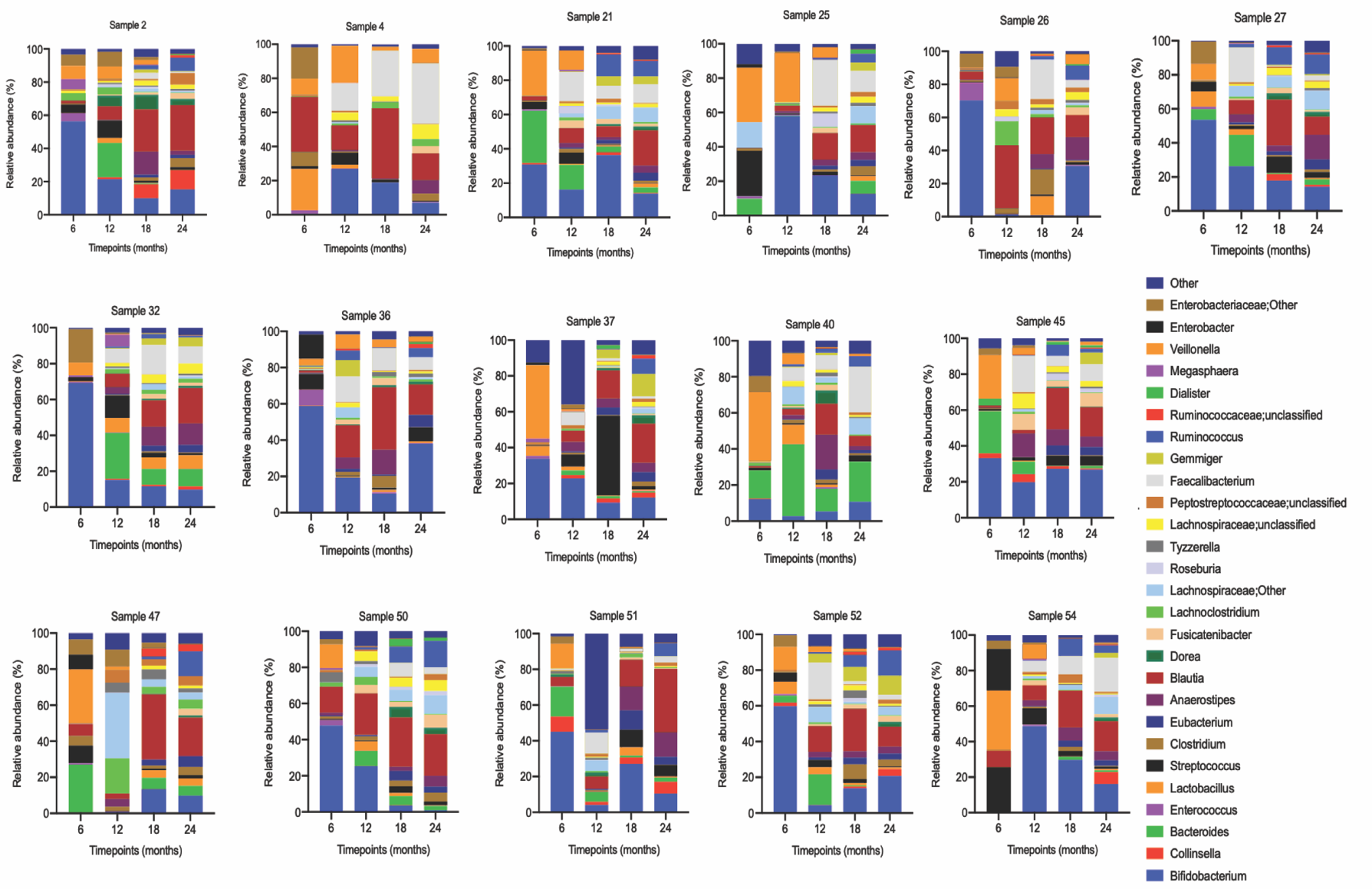
**

**Figure S3**. Relative abundance (%) of 16S rRNA genes at genus level analyzed in fecal samples using Illumina MiSeq. Values < 1% are summarized in the group “Others”. Only samples with all analyzed timepoints are shown here.

**
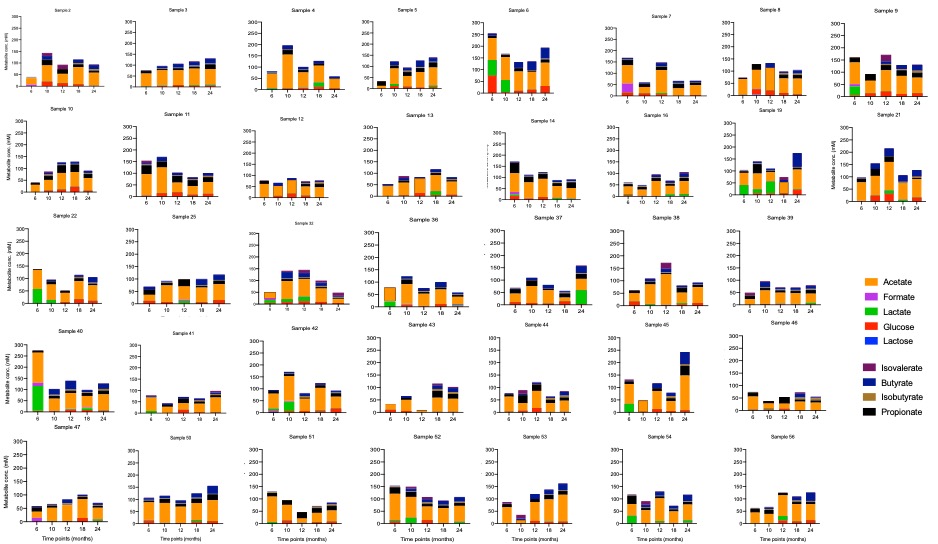
Figure S4**. Metabolite concentrations in fecal samples from 6 to 24 months (n=40) analyzed by HPLC. Only samples with a minimum of 8 analyzed timepoints are shown here.


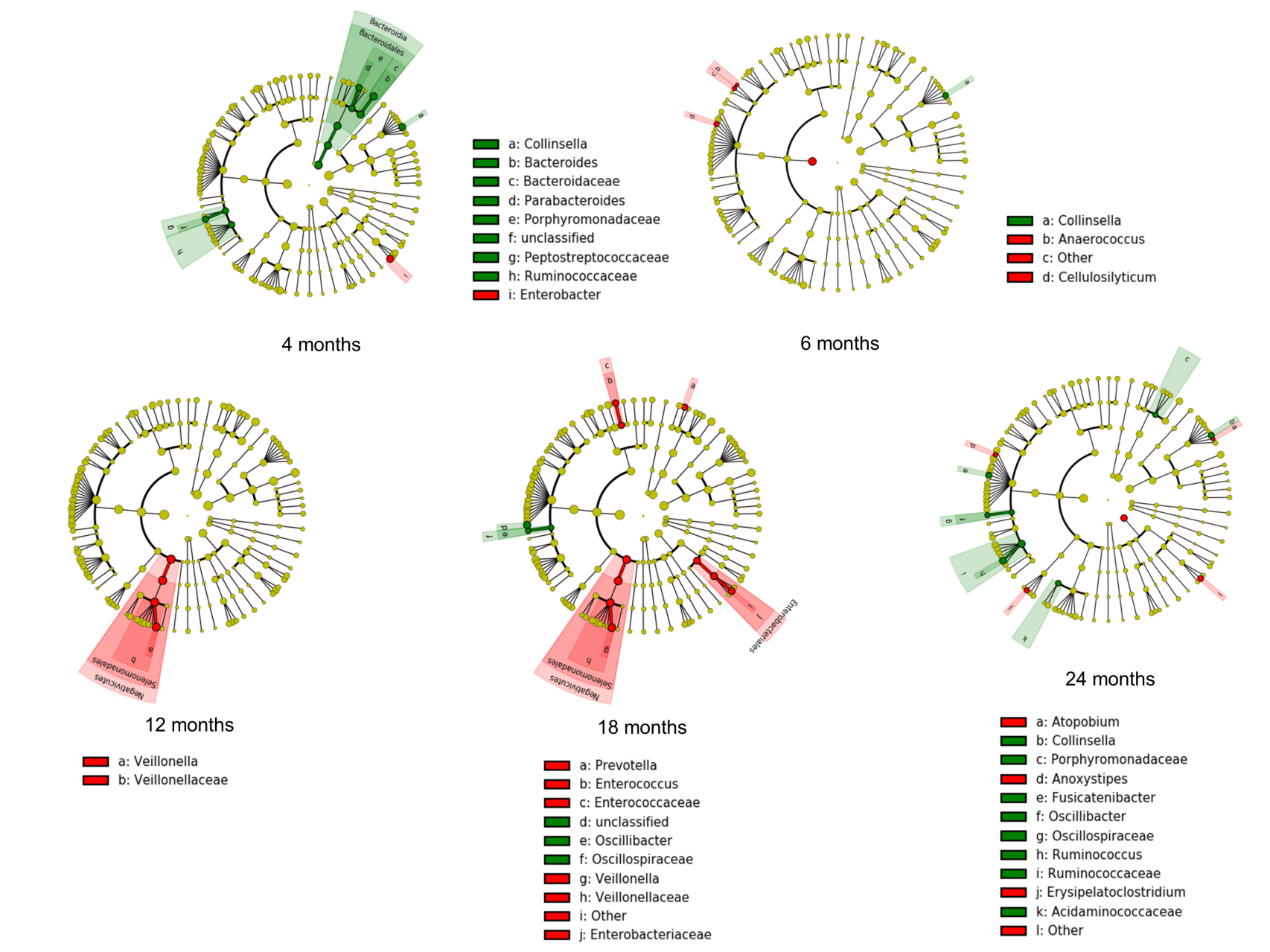
**Figure S5**. Bacterial taxa that were differentially abundant in fecal samples from vaginal delivered infants (green) and Cesarean section delivered infants (red) visualized using a cladogram generated from LEfSe analysis.


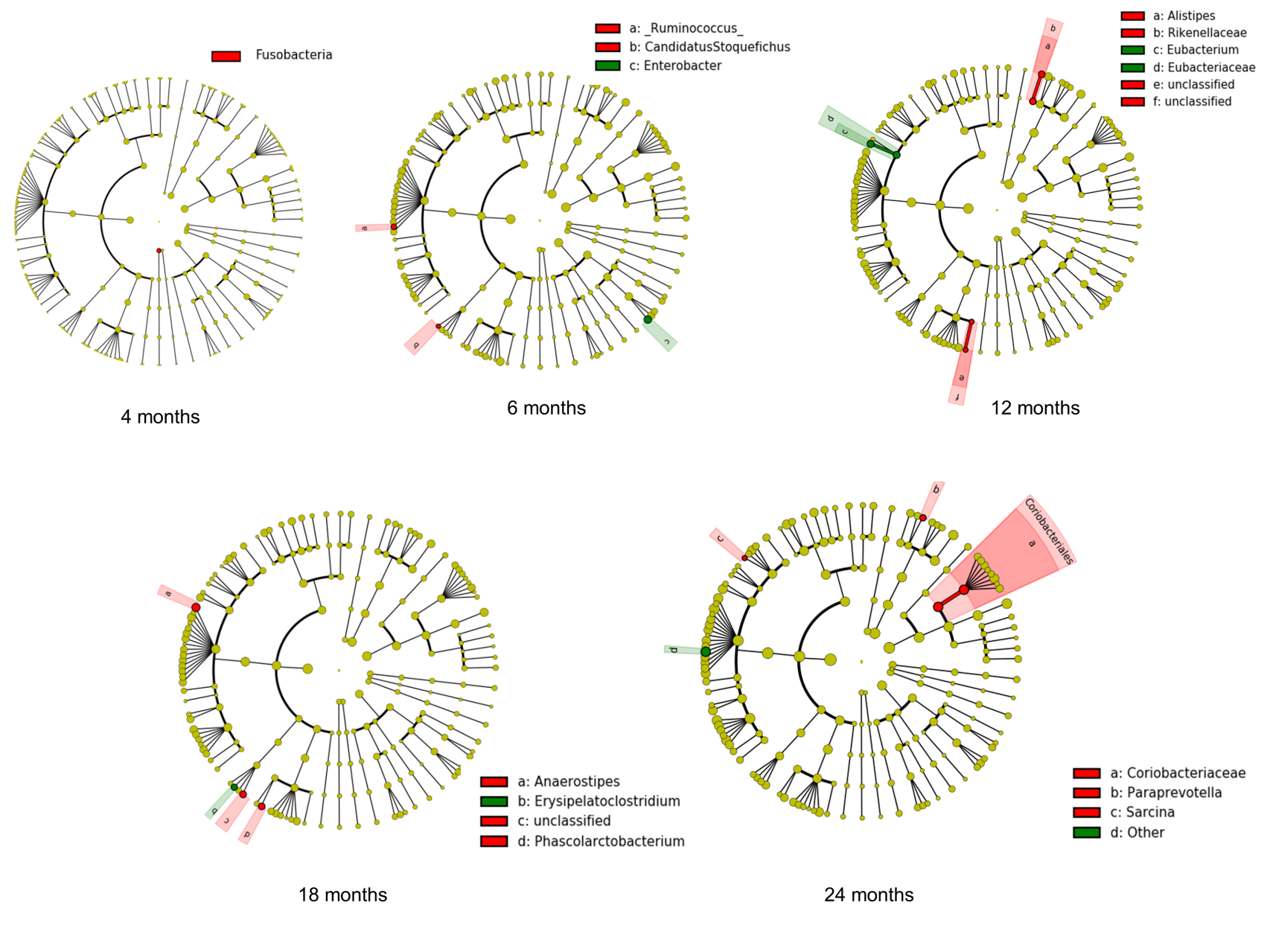
**Figure S6**. Bacterial taxa that were differentially abundant in fecal samples from non-colicky (green) and colicky (red) infants visualized using a cladogram generated from LEfSe analysis

| **Features/ Months** | | **6 m** | **10 m** | **12 m** | **18 m** | **24 m** |
| --- | --- | --- | --- | --- | --- | --- |
| **Gestation (weeks)** | |  |  |  |  |  |
| 37 | | 2 (5) | 2 (5) | 2 (5) | 2 (5) | 2 (5) |
| 38 | | 3 (7.5) | 3 (7.5) | 3 (7.5) | 3 (7.5) | 3 (7.5) |
| 39 | | 11 (27.5) | 11 (27.5) | 11 (27.5) | 11 (27.5) | 11 (27.5) |
| 40 | | 13 (32.5) | 13 (32.5) | 13 (32.5) | 13 (32.5) | 13 (32.5) |
| 41 | | 9 (22.5) | 9 (22.5) | 9 (22.5) | 9 (22.5) | 9 (22.5) |
| 42 | | 2 (5) | 2 (5) | 2 (5) | 2 (5) | 2 (5) |
| **Mode of delivery** | |  |  |  |  |  |
| Caesarean section | | 11 (27.5) | 11 (27.5) | 11 (27.5) | 11 (27.5) | 11 (27.5) |
| Vaginal delivery | | 29 (72.5) | 29 (72.5) | 29 (72.5) | 29 (72.5) | 29 (72.5) |
| **Gender** | |  |  |  |  |  |
| Female | | 22 (55) | 22 (55) | 22 (55) | 22 (55) | 22 (55) |
| Male | | 18 (45) | 18 (45) | 18 (45) | 18 (45) | 18 (45) |
| **Birth weight (kg)** | |  |  |  |  |  |
| >4 | | 3 (7.5) | 3 (7.5) | 3 (7.5) | 3 (7.5) | 3 (7.5) |
| 3.5-4 | | 13 (32.5) | 13 (32.5) | 13 (32.5) | 13 (32.5) | 13 (32.5) |
| 3-3.5 | | 18 (45) | 18 (45) | 18 (45) | 18 (45) | 18 (45) |
| 2.5-3 | | 4 (10) | 4 (10) | 4 (10) | 4 (10) | 4 (10) |
| <2.5 | | 2 (5) | 2 (5) | 2 (5) | 2 (5) | 2 (5) |
| **Antibiotics usage** | |  |  |  |  |  |
| No | | 39 (97.5) | 31 (79.5) | 33 (82.5) | 35 (89.7) | 34 (85) |
| Yes | | 1 (2.5) | 8 (20.5) | 7 (17.5) | 4 (10.3) | 6 (15) |
| **Antibiotics usage of mother** |  |  |  |  |  |  |
| No | | 40 (100) | 39 (100) | 40 (100) | 39 (100) | 40 (100) |
| Yes | | 0 (0) | 0 (0) | 0 (0) | 0 (0) | 0 (0) |
| **Mother milk** | |  |  |  |  |  |
| No | | 34 (87.2) | 37 (92.5) | 39 (100) | 40 (100) | 2 (5) |
| Yes | | 5 (12.8) | 3 (7.5) | 0 (0) | 0 (0) | 38 (95) |
| **Infant formula** | |  |  |  |  |  |
| No | | 2 (5.1) | 8 (20) | 37 (94.9) | 40 (100) | 24 (60) |
| Yes | | 37 (94.9) | 32 (80) | 2 (5.1) | 0 (0) | 16 (40) |
| **Cow milk** | |  |  |  |  |  |
| No | | 31 (79.5) | 15 (37.5) | 2 (5.1) | 0 (0) | 40 (100) |
| Yes | | 8 (20.5) | 25 (62.5) | 37 (94.9) | 40 (100) | 0 (0) |
| **Meat** | |  |  |  |  |  |
| No | | 1 (2.6) | 2 (5) | 2 (5.1) | 2 (5) | 40 (100) |
| Yes | | 38 (97.4) | 38 (95) | 37 (94.9) | 38 (95) | 0 (0) |
| **Yoghurt** | |  |  |  |  |  |
| No | | 10 (25.6) | 5 (12.5) | 1 (2.6) | 2 (5) | 40 (100) |
| Yes | | 29 (74.4) | 35 (87.5) | 38 (97.4) | 38 (95) | 0 (0) |
| **Complimentary food** | |  |  |  |  |  |
| No | | 0 (0) | 0 (0) | 0 (0) | 0 (0) | 40 (100) |
| Yes | | 39 (100) | 40 (100) | 39 (100) | 40 (100) | 0 (0) |

**Table S1**. Baseline characterization and feeding practice of the study population obtained from questionnaires (n=40). Data are given as averaged value (percentage).

**Table S2**. Primers and standards used in this study to enumerate specific bacterial groups by qPCR.

| **Target group** | **Primer** | **Sequence (5’-3’)** | **Standard** | **Detection limit (gene copies/ µl)^1^** | **References** |
| --- | --- | --- | --- | --- | --- |
| Total bacteria | Eub338F  Eub518R | ACT CCT ACG GGA GGC AGC AG  ATT ACC GCG GCT GCTG G | Plasmid pLME21, containing *Bifidobacterium lactis*  16S rDNA | 3.00E+02 | (Guo *et al.*, 2008) |
| Firmicutes | Firm934F  Firm1060R | GGA GYA TGT GGT TTA ATT CGA AGC A  AGC TGA CGA CAA CCA TGC AC | *Roseburia intestinalis* DSM14610T/16S rRNA | 1.00E+02 | (Guo *et al.*, 2008) |
| Enterobacteriaceae | Eco1457F  Eco1652R | CAT TGA CGT TAC CCG CAG AAG AAG C  CTC TAC GAG ACT CAA GCT TGC | *Escherichia coli*  DSM5698/16S rRNA | 5.00E+01 | (Bartosch *et al.*, 2004) |
| *Bacteroides* spp. | Bac303F  Bfr-Fermrev | GAA GGT CCC CCA CAT TG  CGC KAC TTG GCT GGT TCA | *Bacteroides thetaiotaomicron* DSM2079T/16S rRNA | 1.00E+01 | (Ramirez-Farias *et al.*, 2009) |
| *Lactobacillus*/ *Leuconostoc*/ *Pediococccus* spp. | F_Lacto 05  R_Lacto 04 | AGC AGT AGG GAA TCT TCC A  CGC CAC TGG TGT TCY TCC ATA TA | *Lactobacillus delbrueckii* DSM20081T/16S rRNA | 5.00E+01 | (Furet *et al.*, 2009) |
| *Bifidobacterium* spp. | xfp-fw  xfp-rv | ATCTTCGGACCBGAYGAGAC  CGATVACGTGVACGAAGGAC | *Bifidobacterium longum* DSM20219T *xfp^2^* amplicon | 3.00E+03 | (Cleusix *et al.*, 2010) |
| *Streptococcus* spp. | Tuf-Strep-1  Tuf-Strep-R | GAA GAA TTG CTT GAA TTG GTT GAA  GGA CGG TAG TTG TTG AAG AAT GG | *Streptococcus mitis*  DSM12643T/ *tuf*^3^ amplicon | 1.00E+03 | (Collado *et al.*, 2009) |
| *Staphylococcus* spp. | TStaG422  TStag765 | GGC CGT GTT GAA CGT GGT CAA ATC A  TYA CCA TTT CAG TAC CTC TGG TAA | *Staphylococcus epidermidis* DSM20044T/ *tuf*^3^ amplicon | 1.00E+03 | (Martineau *et al.*, 2001) |
| Sulfate-reducing bacteria | dsrA_F336  dsrA_R533 | CTG CGA ATA TGC CTG CTA CA  TGG TCG ARC TTG ATG TCG TC | *Desulfovibrio piger*  DSM 749/ drsA^4^ amplicon | 1.00E+00 | (Pereyra *et al.*, 2010) |
| *Veillonella* | Vpa_X84005_F  Vpa_X84005_R | TGC TAA TAC CGC ATA CGA TCT AAC C  GCT TAT AAA TAG AGG CCA CCT TTC A | *Veillonella parvula*  DSM2008/ 16S rRNA | 1.00E+02 | (Price *et al.*, 2007) |
| *Anaerobutyricum hallii* | EhalF  EhalR | GCG TAG GTG GCA GTG CAA  GCA CCG RAG CCT ATA CGG | *Eubacterium hallii*  DSM3353/ 16S rRNA | 5.00E+01 | (Ramirez-Farias *et al.*, 2009) |
| *Roseburia* spp. | RrecF  Rrec630mR | GCG GTR CGG CAA GTC TGA  CCT CCG ACA CTC TAG TMC GAC | *Roseburia intestinalis* DSM14610T/16S rRNA | 5.00E+01 | (Furet *et al.*, 2009) |
| *Faecalibacterium prausnitzii* | Fprau223F  Fprau420R | GAT GGC CTC GCG TCC GAT TAG  CCG AAG ACC TTC TTC CTC C | *Faecalibacterium prausnitzii* DSM17677/16S rRNA | 5.00E+01 | (Bartosch *et al.*, 2004) |
| Clostridium cluster IV | Clep866mF  Clep1240mR | TTAACACAATAAGTWATCCACCTGG  ACCTTCCTCCGTTTTGTCAAC | *Faecalibacterium prausnitzii* DSM17677/16S rRNA | 5.00E+01 | (Ramirez-Farias *et al.*, 2009) |
| ^1^ qPCR detection limit was defined as the gene copies/ µl of the standard at highest dilution that could generate good amplification and melting curve  ^2^ xylose-5-phosphate/ fructose-6-phosphate phosphoketolase gene  ^3^ elongation factor Tu gene  ^4^ dissimilatory (bi)sulfite reductase gene | | | | | |

**Table S3**. Colonization prevalence (%) of selected bacterial groups detected in feces of infants (n=40) at 6, 8, 10, 12, 15, 18 and 24 months by culture-based and qPCR.

|  | |  | | **Colonization prevalence (%)** | | | | | | | | | | | | | | | |
| --- | --- | --- | --- | --- | --- | --- | --- | --- | --- | --- | --- | --- | --- | --- | --- | --- | --- | --- | --- |
|  | **Bacterial, genus or species** | | | **LPB** | **LUB** | **6 m** | | **8 m** | | | **10 m** | **12 m** | | | **15 m** | | | **18 m** | **24 m** |
| **Culture** | **Total anaerobes** | | | - | - | 100 | | 100 | | | 100 | 100 | | | 100 | | | 100 | 100 |
|  | Cul Total LUB | | |  |  |  | |  | | |  |  | | |  | | |  |  |
|  |  | | Cul LUB SRB | - | + | 97 | | 100 | | | 97 | 92 | | 92 | | | 92 | | 100 |
|  |  | | Cul LUB non-SRB | - | + | 100 | | 97 | | | 100 | 94 | | 100 | | | 100 | | 100 |
| **qPCR** | **Total bacteria** | | | - | - | 100 | | n.a | | | 100 | 100 | | | n.a | | | 100 | 100 |
|  | Firmicutes | | | - | - | 100 | | n.a | | | 100 | 100 | | | n.a | | | 100 | 100 |
|  | Enterobacteriaceae | | | - | - | 100 | | n.a | | | 100 | 100 | | | n.a | | | 100 | 100 |
|  | *Bacteroides* | | | + | - | 95 | | n.a | | | 100 | 100 | | | n.a | | | 100 | 100 |
|  | *Lactobacillus* | | | + | - | 92 | | n.a | | | 100 | 100 | | | n.a | | | 100 | 100 |
|  | *Bifidobacterium* | | | + | - | 100 | | n.a | | | 100 | 100 | | | n.a | | | 100 | 100 |
|  | *Streptococcus* | | | + | - | 59 | | n.a | | | 100 | 100 | | | n.a | | | 100 | 100 |
|  | *Staphylococcus* | | | + | - | 21 | | n.a | | | 65 | 83 | | | n.a | | | 50 | 30 |
|  | **qPCR Total LUB** | | |  |  |  | |  | | |  |  | | |  | | |  |  |
|  |  | | SRB | - | + | 23 | | n.a | | | 13 | 15 | | n.a | | | 18 | | 33 |
|  |  | | *Veillonella* | - | + | 100 | | n.a | | | 100 | 100 | | n.a | | | 100 | | 100 |
|  |  | | *Anaerobutyricum hallii* | - | + | 13 | | n.a | | | 25 | 40 | | n.a | | | 83 | | 85 |
|  | **qPCR Total BPB** | | |  |  |  |  | |  |  | | |  | | |  |  | | |
|  |  | | *Anaerobutyricum hallii* | - | + | 13 | | n.a | | | 25 | 40 | | n.a | | | 83 | | 85 |
|  |  | | *Roseburia* | - | - | 41 | | n.a | | | 90 | 100 | | n.a | | | 100 | | 100 |
|  |  | | *Clostridium Cluster IV* | + | - | 69 | | n.a | | | 100 | 100 | | n.a | | | 100 | | 100 |
|  |  | | *Faecalibacterium prausnitzii* | + | - | 54 | | n.a | | | 100 | 100 | | n.a | | | 100 | | 100 |
| Cul, culturable; LPB, lactate-producing bacteria; LUB, lactate-utilizing bacteria; SRB, sulfate-reducing bacteria.  n.a. = not analyzed. | | | | | | | | | | | | | | | | | | | |

**Table S4**. Prevalence of sugars (lactose, glucose), intermediate metabolites (lactate, formate), SCFA (acetate, propionate and butyrate) and BCFA (isobutyrate and isovalerate) detected in feces of infants (n=40) from 6 to 24 months.

|  |  |  | **Prevalence (%)** | | | | |
| --- | --- | --- | --- | --- | --- | --- | --- |
| **Metabolites** |  |  | **6 m** | **10 m** | **12 m** | **18 m** | **24 m** |
| **Sugars** | | | | | | | |
|  |  | **Lactose** | 8 | 5 | 8 | 3 | 3 |
|  |  | **Glucose** | 65 | 97 | 97 | 100 | 100 |
| **Intermediate metabolites** | | | | | | | |
|  |  | **Lactate** | 60 | 44 | 28 | 38 | 35 |
|  |  | **Formate** | 20 | 3 | 3 | 3 | 3 |
| **SCFA** | | | | | | | |
|  |  | **Acetate** | 100 | 100 | 100 | 100 | 100 |
|  |  | **Propionate** | 95 | 92 | 92 | 98 | 100 |
|  |  | **Butyrate** | 73 | 87 | 82 | 100 | 100 |
| **BCFA** | | | | | | | |
|  |  | **Isobutyrate** | 53 | 59 | 62 | 95 | 98 |
|  |  | **Isovalerate** | 48 | 62 | 54 | 75 | 78 |

**Table S5:** Changes over the time course from 6 to 24 months in levels of functional groups of microbes in infant feces (n=40) using culture-based methods and qPCR (A) and metabolite concentrations (B) using linear regression model including data from all timepoints.

| **A.** | | **Bacterial, genus or species** | | | **Beta** | **Sig.** |
| --- | --- | --- | --- | --- | --- | --- |
| **Culture** | | **Total anaerobes** | | | -0.042 | 0.294 |
|  | | Cul Total LUB | | |  |  |
|  | |  | Cul LUB SRB | | 0.038 | 0.427 |
|  | |  | Cul LUB non-SRB | | 0.024 | 0.594 |
| **qPCR** | | **Total bacteria** | | | -0.057 | 0.563 |
|  | | Firmicutes | | | 0.226 | **0.002** |
|  | | Enterobacteriaceae | | | -0.103 | **0.046** |
|  | | *Bacteroides* | | | 0.025 | 0.685 |
|  | | *Lactobacillus* | | | -0.099 | **0.034** |
|  | | *Bifidobacterium* | | | 0.059 | 0.322 |
|  | | *Streptococcus* | | | 0.027 | 0.623 |
|  | | *Staphylococcus* | | | -0.180 | **0.006** |
|  | | **qPCR Total LUB** | | |  |  |
|  | |  | SRB | | -0.006 | 0.889 |
|  | |  | *Veillonella* | | -0.169 | **0.026** |
|  | |  | *Anaerobutyricum hallii* | | 0.152 | **0.036** |
|  | | **qPCR Total BPB** | | |  |  |
|  | |  | *Anaerobutyricum hallii* | | 0.152 | **0.036** |
|  | |  | *Roseburia* | | 0.102 | 0.068 |
|  | |  | *Clostridium Cluster IV* | | 0.137 | 0.132 |
|  | |  | *Faecalibacterium prausnitzii* | | -0.144 | 0.072 |
|  | |  |  | |  |  |
| **B.** | |  |  |  | |  |
| **Metabolites** | |  | **Beta** | **Sig.** | |  |
| **Lactose** | |  | -0.042 | 0.324 | |  |
| **Glucose** | |  | 0.179 | **0.001** | |  |
| **Lactate** | |  | 0.072 | 0.271 | |  |
| **Formate** | |  | -0.073 | 0.205 | |  |
| **Acetate** | |  | -0.122 | 0.062 | |  |
| **Propionate** | |  | -0.009 | 0.851 | |  |
| **Butyrate** | |  | 0.334 | **0.000** | |  |
| **Isobutyrate** | |  | 0.077 | 0.169 | |  |
| **Isovalerate** | |  | 0.005 | 0.916 | |  |

**Table S6.** Comparison of metabolite concentrations of non-colicky (n=32) and colicky (n=8) and Caesarean born (CS) (n=11) versus vaginally delivered (VD) (n=29) infants from 6 months to 24 months. Values are expressed as means ± SD mM. Means were compared pairwise using Student’s t-test for normally distributed data. Non-parametric Mann-Whitney test was performed when data were not normally distributed. P values< 0.05 were considered significant.

|  | 6 months | | |  | 10 months | | |  | 12 months | | |  | 18 months | | |  | 24 months | | |
| --- | --- | --- | --- | --- | --- | --- | --- | --- | --- | --- | --- | --- | --- | --- | --- | --- | --- | --- | --- |
|  | Non-colic | Colic | P |  | Non-colic | Colic | P |  | Non-colic | Colic | P |  | Non-colic | Colic | P |  | Non-colic | Colic | P |
| Lactose | 0.16 ± 0.92 | 0.12 ± 0.33 | 0.80 |  | 0.02 ± 0.13 | 0.50 ± 1.41 | 0.68 |  | 0.04 ± 0.14 | 0.00 ± 0.00 | 0.70 |  | 0.00 ± 0.00 | 0.22 ± 0.61 | 0.61 |  | 0.02 ± 0.10 | 0.00 ± 0.00 | 0.91 |
| Glucose | 7.07 ± 13.41 | 1.19 ± 1.69 | 0.08 |  | 5.97 ± 4.76 | 8.61 ± 8.47 | 0.75 |  | 8.51 ± 6.66 | 12.15 ± 5.16 | 0.07 |  | 7.31 ± 5.17 | 5.94 ± 2.39 | 0.65 |  | 7.70 ± 6.67 | 4.75 ± 2.79 | 0.43 |
| Lactate | 13.81 ± 24.92 | 5.97 ± 11.50 | 0.50 |  | 5.59 ± 11.84 | 0.76 ± 1.44 | 0.40 |  | 4.1 ± 9.88 | 0.00 ± 0.00 | 0.14 |  | 2.99 ± 5.10 | 0.26 ± 0.75 | 0.17 |  | 3.84 ± 10.01 | 0.00 ± 0.00 | 0.06 |
| Formiate | 2.98 ± 7.86 | 0.84 ± 2.38 | 0.65 |  | 0.12 ± 0.68 | 0.00 ± 0.00 | 0.91 |  | 0.14 ± 0.80 | 0.00 ± 0.00 | 0.91 |  | 0.13 ± 0.74 | 0.00 ± 0.00 | 0.91 |  | 0.15 ± 0.87 | 0.00 ± 0.00 | 0.91 |
| Acetate | 56.63 ± 28.29 | 64.07 ± 21.66 | 0.49 |  | 64.79 ± 27.91 | 45.25 ± 23.88 | 0.08 |  | 63.32 ± 26.28 | 51.66 ± 28.53 | 0.28 |  | 57.09 ± 16.5 | 51.71 ± 20.6 | 0.44 |  | 58.08 ± 17.8 | 69.20 ± 37.0 | 0.65 |
| Propionate | 14.46 ± 11.03 | 9.74 ± 5.56 | 0.45 |  | 15.84 ± 8.34 | 10.31 ± 10.37 | 0.12 |  | 12.88 ± 8.96 | 14.25 ± 10.33 | 0.71 |  | 12.89 ± 7.47 | 13.55 ± 3.92 | 0.81 |  | 14.29 ± 6.37 | 16.86 ± 9.83 | 0.61 |
| Isobutyrate | 1.06 ± 1.25 | 1.19 ± 1.41 | 0.91 |  | 1.78 ± 1.45 | 0.00 ± 0.00 | **0.00** |  | 2.29 ± 1.86 | 0.00 ± 0.00 | **0.00** |  | 3.48 ± 1.82 | 5.43 ± 1.85 | **0.01** |  | 3.78 ± 1.88 | 5.56 ± 1.53 | **0.02** |
| Butyrate | 3.18 ± 3.16 | 1.76 ± 1.34 | 0.43 |  | 10.05 ± 6.89 | 5.68 ± 6.86 | 0.12 |  | 11.79 ± 8.68 | 9.74 ± 9.31 | 0.68 |  | 13.34 ± 7.69 | 11.00 ± 5.83 | 0.50 |  | 18.43 ± 12.44 | 16.64 ± 15.48 | 0.38 |
| Isovalerate | 1.25 ± 2.69 | 2.41 ± 2.10 | **0.02** |  | 2.9 ± 3.87 | 3.49 ± 6.48 | 0.24 |  | 3.35 ± 6.46 | 2.50 ± 7.07 | 0.06 |  | 2.05 ± 2.70 | 1.88 ± 1.63 | 0.75 |  | 2.03 ± 2.55 | 2.89 ± 2.16 | 0.13 |
|  | CS | VD | P |  | CS | VD | P |  | CS | VD | P |  | CS | VD | P |  | CS | VD | P |
| Lactose | 1.04 + 2.33 | 0.05 + 0.22 | 0.75 |  | 0.00 + 0.00 | 0.33 + 1.15 | 0.86 |  | 0.00 + 0.00 | 0.00 + 0.00 | 1.00 |  | 0.00 + 0.00 | 0.13 + 0.48 | 0.90 |  | 0.00 + 0.00 | 0.04 + 0.15 | 0.90 |
| Glucose | 1.72 + 2.08 | 3.49 + 3.06 | 0.23 |  | 6.42 + 9.12 | 9.14 + 7.84 | 0.57 |  | 13.66 + 2.88 | 13.39 + 8.71 | 0.95 |  | 4.73 + 2.83 | 6.49 + 4.16 | 0.50 |  | 3.1 + 0.49 | 7.5 + 5.05 | **0.01** |
| Lactate | 3.11 + 4.28 | 15.06 + 28.93 | 0.49 |  | 0.00 + 0.00 | 1.66 + 4.03 | 0.52 |  | 0.00 + 0.00 | 2.6 + 6.14 | 0.68 |  | 0.00 + 0.00 | 1.69 + 4.7 | 0.70 |  | 0.00 + 0.00 | 0.00 + 0.00 | 1.00 |
| Formiate | 1.35 + 3.01 | 2.36 + 4.75 | 0.91 |  | 0.00 + 0.00 | 0.00 + 0.00 | 1.00 |  | 0.00 + 0.00 | 0.00 + 0.00 | 1.00 |  | 0.00 + 0.00 | 0.00 + 0.00 | 1.00 |  | 0.00 + 0.00 | 0.00 + 0.00 | 1.00 |
| Acetate | 55.27 + 15.58 | 67.05 + 30.73 | 0.42 |  | 35.56 + 24.6 | 57.96 + 23.63 | 0.13 |  | 54 + 22.56 | 58.68 + 34.12 | 0.80 |  | 48.76 + 22 | 53.77 + 15.9 | 0.70 |  | 54.88 + 10.6 | 60.48 + 27.1 | 1.00 |
| Propionate | 7.72 + 4.69 | 13.58 + 9.85 | 0.22 |  | 15.77 + 14.61 | 14.63 + 9.98 | 0.86 |  | 19.18 + 5.61 | 12.87 + 11.23 | 0.17 |  | 11.95 + 1.78 | 14.3 + 7.6 | 0.61 |  | 10.79 + 2.12 | 15.58 + 9.04 | 0.52 |
| Isobutyrate | 0.56 + 0.81 | 1.14 + 1.43 | 0.54 |  | 0.00 + 0.00 | 0.35 + 1.21 | 0.86 |  | 0.00 + 0.00 | 0.64 + 1.55 | 0.68 |  | 5.56 + 0.95 | 5.69 + 1.49 | 0.89 |  | 5.93 + 1.11 | 5.78 + 1.38 | 0.86 |
| Butyrate | 3.08 + 1.45 | 2.12 + 2.42 | 0.11 |  | 9.3 + 5.1 | 6.74 + 8.22 | 0.52 |  | 9.04 + 8.5 | 9.19 + 10.6 | 0.86 |  | 9.73 + 5 | 11.76 + 6.37 | 0.62 |  | 13.58 + 6.57 | 17.38 + 13.5 | 0.90 |
| Isovalerate | 1.89 + 2.7 | 1.42 + 2.68 | 0.64 |  | 10.7 + 7.2 | 1.09 + 2.5 | **0.04** |  | 5.92 + 9.55 | 2.24 + 7.76 | 0.32 |  | 1.24 + 1.12 | 1.93 + 2.22 | 0.90 |  | 4.66 + 2.46 | 1.56 + 1.6 | **0.04** |
|  |  |  |  |  |  |  |  |  |  |  |  |  |  |  |  |  |  |  |  |
